# Aging disrupts spatiotemporal regulation of germline stem cells and niche integrity

**DOI:** 10.1242/bio.060261

**Published:** 2024-01-15

**Authors:** Michelle A. Urman, Nimmy S. John, Tyler Jung, ChangHwan Lee

**Affiliations:** ^1^ Department of Biological Sciences, University at Albany, State University of New York, Albany, NY, 12222, USA; ^2^ The RNA Institute, University at Albany, State University of New York, Albany, NY, 12222, USA

**Keywords:** *Caenorhabditis elegans* gonad, Germline stem cells, Aging, Notch signaling, Transcriptional regulation, Spatial pattern analysis, *sygl-1*, Gradient

## Abstract

A major factor driving stem cell decline is stem cell niche aging, but its molecular mechanism remains elusive. We use the *Caenorhabditis elegans* distal tip cell (DTC), the mesenchymal niche that employs Notch signaling to regulate germline stem cells (GSCs), as an *in vivo* niche aging model and delineate the molecular details of the DTC/niche aging process. Here, we demonstrate that a drastic decrease in *C. elegans* germline fecundity, which begins even in early adulthood, is mainly due to an age-induced disruption in spatial regulation of Notch-dependent transcription in the germline combined with a moderate reduction in Notch transcription at both tissue and cellular levels. Consequently, the Notch-responsive GSC pool shifts from the distal end of the gonad to a more proximal region, disrupting the distal-to-proximal germline polarity. We find that this GSC pool shift is due to a dislocation of the DTC/niche nucleus, which is associated with age-induced changes in the structure and morphology of the DTC/niche. Our findings reveal a critical link between physiological changes in the aging niche, their consequences in stem cell regulation, and germline tissue functions.

## Teaser

**Summary:** Aging disrupts spatial regulation of Notch activation in the *C. elegans* germline, leading to the mislocation of the Notch-responsive GSC pool, leading to reproductive decline, which is mainly due to an age-associated DTC/niche nuclear shift and its structural changes.

## INTRODUCTION

Aging is characterized by degenerative changes and progressive declines in cellular and tissue functions (Harman, 1981; Kocsisova et al., 2019). Elucidating the aging process has been a challenge due to its complexity, as aging is a result of a multitude of intrinsic and extrinsic factors interplaying, such as DNA damage, accumulation of toxic metabolites, and environmental stresses (Oh et al., 2014). Aging can occur at different biological levels, including molecular, cellular, and organismal levels, exhibiting age-induced physiological defects such as protein damage, cellular disintegration, or tumorigenesis, which increases the susceptibility to age-related diseases like neurodegeneration and cancer (Booth and Brunet, 2016; DiLoreto and Murphy, 2015; Johnson et al., 1999; Mc Auley et al., 2017).

Age-induced tissue loss or decline has been attributed to a decrease in adult stem cell function (Brunet et al., 2022; Oh et al., 2014; Wallenfang, 2007). Stem cells can either remain activated or become quiescent upon the completion of development, in both cases exhibiting a progressive decline in their function and frequency during aging (Charville and Rando, 2011; Devaraj et al., 2021; Pan et al., 2007; Rossi et al., 2008). However, recent studies suggest that stem cells age minimally intrinsically, and extrinsic factors such as microenvironments are largely responsible for age-induced stem cell decline (Kalamakis et al., 2019; Morrow and Moore, 2019). This indicates that biological aging of stem cells is different from its chronological aging. Unraveling the molecular mechanisms of biological stem cell aging will advance our knowledge to delay tissue deterioration, reverse the aging process, and alleviate age-related diseases.

A major extrinsic factor that regulates stem cell function is the stem cell niche, which is located adjacent to stem cells and provides their microenvironment. Since niches are typically specialized somatic cells, they also experience biological aging, including a structural or functional decline (Jasper and Kennedy, 2012; Morrow and Moore, 2019; Ryu et al., 2006; Wallenfang, 2007). For example, the aging stem cell niche in the female *Drosophila* gonad progressively loses physical contact with its stem cells (Pan et al., 2007), and signaling from the neural stem cell niche weakens with aging, gradually losing the stem cells that can exit the quiescent state upon injury (Kalamakis et al., 2019; Morrow and Moore, 2019). Niche aging has been suggested to be a major driver of age-induced stem cell decline (Brunet et al., 2022; Goodell and Rando, 2015; Navarro Negredo et al., 2020), but the molecular mechanisms of niche aging and how it affects stem cell physiology and tissue homeostasis remain poorly understood.

**Fig. 1.**
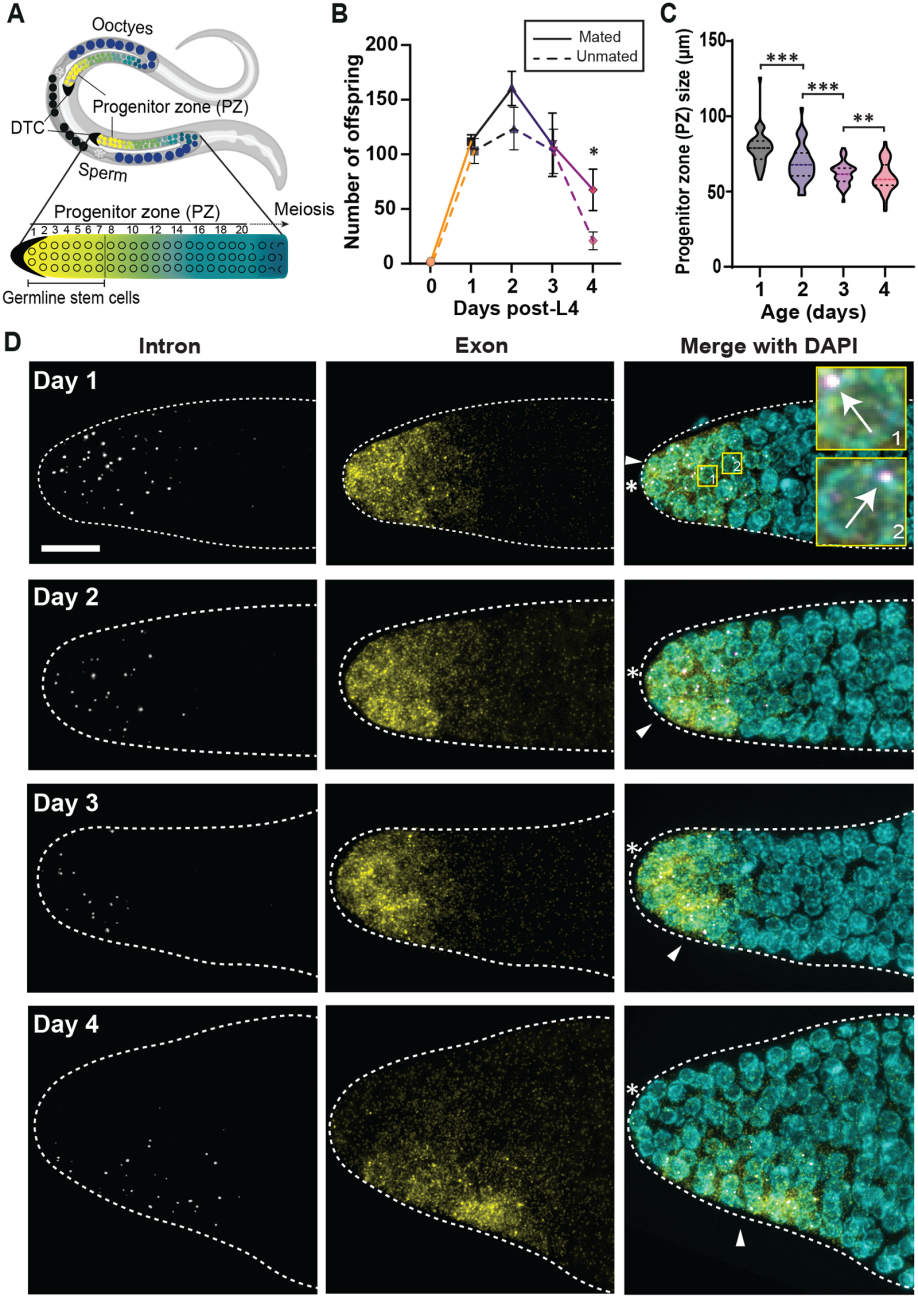
**Age-induced decline in germline function begins early in adulthood whereas the germ cells age minimally.** (A) Schematic of adult *C. elegans* hermaphrodite with U-shaped gonads with a distal tip cell (DTC) at each distal end (black crescents). Along the proximal end, oocytes (dark blue) reside that meet with sperm to form eggs (black). Germline stem cells (GSCs; yellow) occupy the first 6–8 germ cell rows of the germline. The GSCs progress through the progenitor zone, begin differentiation (green) to oocytes by entering meiosis (blue). (B) Egg laying rate for mated (solid line) and unmated (dashed line) N2 hermaphrodites from mid-L4 to Day 4. (C) The size of the progenitor zone (PZ), which measures the distance between the distal end of the gonad and the first cells in meiotic prophase (crescents). *n*=45 gonads. (B,C) All error bars in this study are the standard error of the mean (SEM) unless stated otherwise. For all *t*-tests (one sample or two sample) in this study, *P<0.05, **P<0.01, ***P<0.001, and ****P<0.0001. ‘n.s.’: not significant by *t*-test. (D) *sygl-1* smFISH with the wild type, N2, at different aging stages. Day 1: the young adult stage (24h post mid-L4), Day 2: 48h post L4, Day 3: 72h post L4, and Day 4: 96h post L4. The distal gonads are shown in Z-projection. Left: the intron probes reveal *sygl-1* nascent transcripts at the active transcription sites (ATS). Middle: the exon probes show both the nascent transcripts (bright dots) and cytoplasmic mRNAs (small, dim dots). Right: DAPI marks DNA in the nucleus. The arrow indicates ATS, which is seen both in the intron and exon channel. White asterisk: distal end of the gonad. Arrowhead: the position of the DTC/niche nucleus. Scale bar: 10 µm, the area in the numbered yellow boxes are shown 10× zoomed in on the right.

Niches regulate stem cells using intercellular signaling, such as Notch, Wnt, or Hedgehog signaling (Wang et al., 2009). These pathways are often highly contextual, with their activation timing, duration, and targets varying depending on the tissue type, developmental stage, or environmental conditions (Carlson et al., 2008; Housden and Perrimon, 2014). Therefore, analyzing niche signaling in its native contexts is crucial to precisely understand *in vivo* niche regulation on stem cells and their aging process. Here, we use the *Caenorhabditis elegans* gonad as an *in vivo* niche aging model, where the mesenchymal distal tip cell (DTC) serves as a niche for a pool of 35–70 germline stem cells (GSCs) (Crittenden et al., 1994, 2006; Kimble and Crittenden, 2007; Lee et al., 2016) (Fig. 1A). The DTC/niche, located at the distal end of the gonad, is essential for germline tissue organization, GSC regulation, and gametogenesis (Austin and Kimble, 1987; Byrd and Kimble, 2009; Byrd et al., 2014; Cinquin et al., 2010; Crittenden et al., 1994; Kimble and Crittenden, 2007; Kimble and White, 1981). The DTC/niche is composed of a cap with elaborate cellular processes that enwrap the distal gonad and infiltrate into the GSC pool to ensure physical interactions with the GSCs for niche signaling transduction (Linden et al., 2017; Wong et al., 2013).

The DTC/niche regulates GSCs using Notch signaling, a broadly conserved cell–cell signaling pathway that activates important biological programs such as cell fate decision, cell proliferation, apoptosis, and tissue patterning (Byrd and Kimble, 2009; Crittenden et al., 2006; Kershner et al., 2014). Dysfunction of Notch often leads to various diseases including cardiovascular disease and cancer (Bocci et al., 2020; Kopan, 2012; Siebel and Lendahl, 2017). Notch is activated upon ligand-receptor interaction, which triggers a series of proteolytic cleavages on the Notch receptor (GLP-1 or LIN-12 in *C. elegans*) and releases the Notch intracellular domain (NICD) that translocates into the nucleus and activates transcription from targets with a DNA-binding protein, CSL/RBPJ-κ, and a co-activator, MAML (LAG-1 and LAG-3/SEL-8 in *C. elegans*, respectively) (Bray, 2006; Chen et al., 2020; Kershner et al., 2014). In the *C. elegans* gonad, GLP-1/Notch activates at least two targets, *sygl-1* and *lst-1*, encoding functionally redundant stem cell effectors (Brenner and Schedl, 2016; Chen et al., 2020; Kershner et al., 2014; Lee et al., 2016; Singh et al., 2011). Nascent transcripts from the active transcription sites (ATS) of the two targets have been established as direct readouts of Notch activation, which revealed a stochastic yet probabilistic nature of Notch signaling (Crittenden et al., 2019; Lee et al., 2016, 2019; Lynch et al., 2022). Importantly, the distribution of *sygl-1* ATS and mRNAs coincides with their protein locations as well as the location and extent of the GSC pool, thus has been considered as a GSC marker (Cinquin et al., 2010; Crittenden et al., 2019; Kershner et al., 2014; Lee et al., 2016, 2019; Shin et al., 2017).

Despite extensive studies delineating Notch functions, little is known about the link between niche aging and Notch activation. Here, we elucidate the DTC/niche aging process and analyze its effects on Notch activation and GSC function in aging *C. elegans*, from its young adult stage (24 h post the mid-L4 larval stage or Day 1) to 96 h post-L4 (Day 4), at which the worms exhaust their premade sperm supply and conclude self-fertility (Andux and Ellis, 2008; Byerly et al., 1976; Kocsisova et al., 2019; Scharf et al., 2021). We show that a significant decline in the mitotic germ cell pool and fecundity, which begins in early adulthood, is not because of the reduction of Notch-dependent transcriptional activation in the germline but mainly due to an age-induced disruption in spatial regulation of Notch-dependent transcription. We observe that the Notch-responsive GSC pool shifts from the distal end of the gonad to a more proximal region with aging, which in turn disrupts the germline tissue polarity, causing premature differentiation of the GSCs at the distal gonad. This GSC shift occurs in close proximity to the DTC/nucleus, which is also mislocated with aging along with changes in the structure and morphology of the DTC/niche. Our results elucidate the molecular details of DTC/niche aging and its consequences on stem cells and tissue function and reveal a correlation between the physiological changes of the DTC/niche and the spatiotemporal regulation of stem cells.

## RESULTS

### The aging process begins in early adulthood and induces a reduction of germ cell progenitor zone (PZ) and fecundity

To elucidate the molecular mechanism of niche aging and its effects on stem cells *in vivo*, this study uses the *C. elegans* gonad, where a single mesenchymal cell, the distal tip cell (DTC) maintains 35–70 germline stem cells (GSCs) at the distal gonad under normal conditions (Cinquin et al., 2010; Crittenden et al., 2017) (Fig. 1A). *C. elegans* hermaphrodites normally produce eggs from young adult (Day 1; 24 h post the L4-larval stage) to 72 h post Day 1 (Day 4) before they exhaust self-made sperm while mated worms typically have an extended fertile period (Pickett et al., 2013; Tolkin and Hubbard, 2021). As previously reported, we observed a sharp decrease in the number of offspring after Day 2 both in the mated and unmated worms (Fig. 1B) (Pickett et al., 2013). Consistently, the progenitor zone (PZ), which harbors the mitotic germ cells including GSCs at the distal end, decreased progressively from Day 1-4 (Fig. 1C) (Kocsisova et al., 2019), indicating that the gonad experiences a functional decline early in adulthood.

### ***sygl-1*** smFISH reveals marginal changes in overall Notch activation in the germline during aging

To precisely understand how the germline tissue ages, we localized the GSCs in the gonad from Days 1–4 and examined their activity and function. Specifically, we performed single-molecule fluorescence *in situ* hybridization (smFISH) to visualize the active transcription sites (ATS) and mRNAs from a well-characterized Notch target, *sygl-1* (Fig. 1D) (Kershner et al., 2014; Lee et al., 2016). *sygl-1* smFISH provides direct Notch readouts to quantify Notch-dependent transcriptional activation, which has been used to identify GSCs and gauge their Notch response (Crittenden et al., 2019; Kershner et al., 2014; Lee et al., 2016, 2022; Lynch et al., 2022; Shin et al., 2017; Sorensen et al., 2020). We conducted smFISH with two probe sets, one targeting *sygl-1* introns and the other targeting exons, which revealed both the nuclear nascent transcripts at *sygl-1* ATS and cytoplasmic *sygl-1* mRNAs at the single-molecule level (Fig. 1D). The intensities of the detected mRNA spots make a very tight distribution for all age groups, the gold standard of single-molecule detection, whereas the ATS spots, consisting of many nascent transcripts, show a much broader range in their intensities as expected (Fig. S1A,B) (Lee et al., 2016; Raj and van Oudenaarden, 2009).

**Fig. 2.**
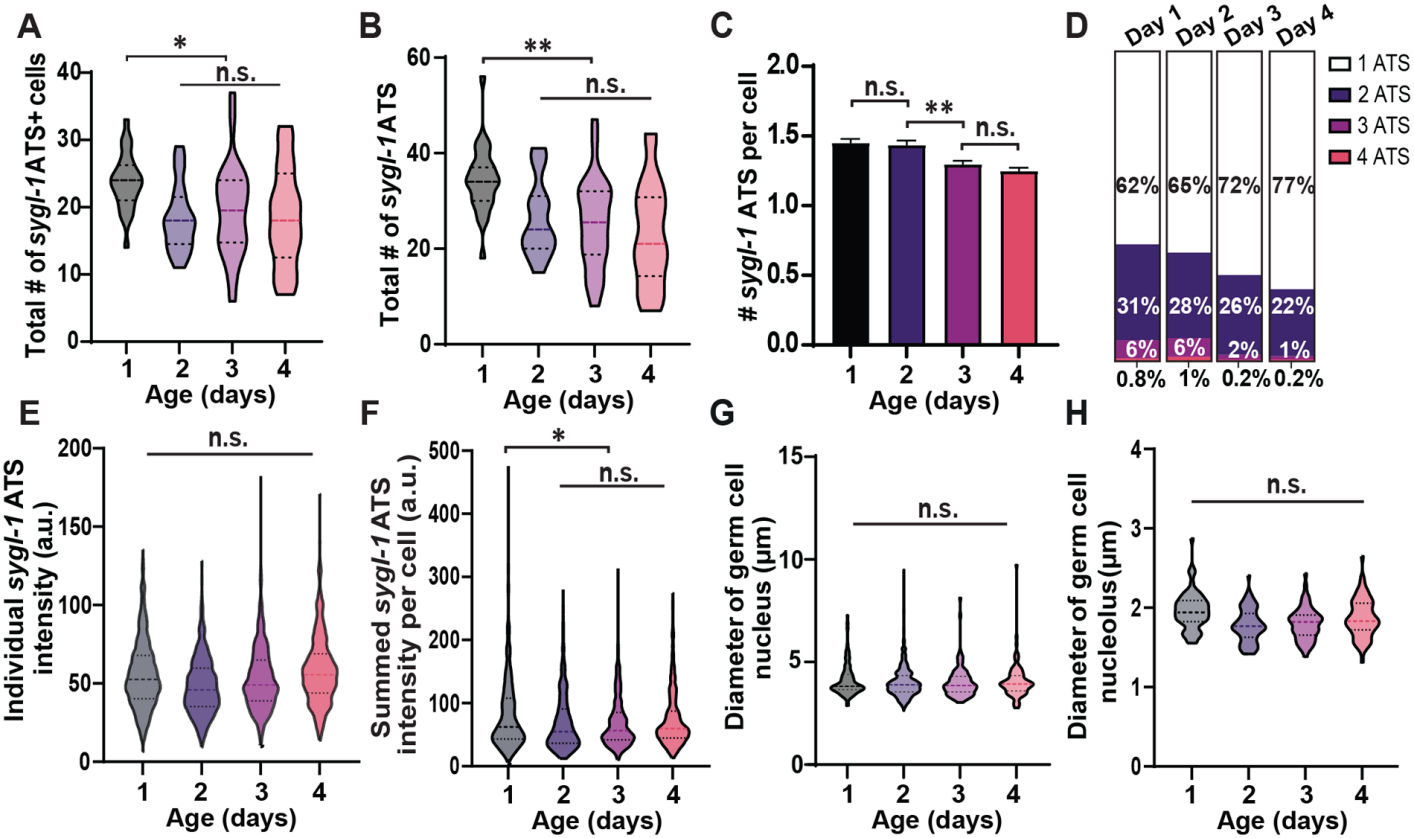
**Aging reduces *sygl-1* transcriptional activation both at the tissue and cellular levels.** (A) The total number of *sygl-1* ATS-containing cells in the gonad for each aging stage. For violin plots in this study, the middle line shows the median; top and bottom dashed lines are the third and first quartiles. (B) The total number of *sygl-1* ATS in the gonad. (C) The average number of *sygl-1* ATS in each germ cell for all ages. (D) The *sygl-1* ATS-containing cells are grouped by the number of ATS they contain and plotted as the percentages for each age. (A–D) *n*=27, 25, 26, and 24 gonads for Day 1, 2, 3, and 4 respectively (E) The individual *sygl-1* ATS intensities are compared between all ages. *n*=621, 460, 508, and 423 nuclei for Day 1, 2, 3, and 4 respectively. (F) The signal intensities of all *sygl-1* ATS in each germ cell are pooled to calculate the summed *sygl-1* ATS intensity, which estimates the overall *sygl-1* transcriptional activity in each cell. *n*=931, 661, 660, and 550 ATS for Day 1, 2, 3, and 4 respectively. (E,F) a.u.: arbitrary units. (G) The diameter of the germ cells located at the distal gonad (0–40 μm from the distal end of the gonad) was measured for each aging stage. *n*=3043, 2645, 2703, and 2662 nuclei for Day 1, 2, 3, and 4 respectively. (H) The diameter of the nucleolus in each germ cell located at the distal gonad (0–40 μm from the distal end) was recorded for all ages. *n*=65 nucleoli for all ages.

*sygl-1* smFISH with aging gonads revealed that Notch-dependent transcription remains active through aging, with many *sygl-1* ATS and mRNAs present even at Day 4 (Fig. 1D), confirming the DTC/niche-GSC interactions in all age groups. We hypothesized that a drastic decline in the PZ and fecundity in aged worms is due to a decreased Notch activation and a GSC loss. To test the idea, we compared the number of Notch-responsive GSCs and their Notch-dependent transcriptional activity across the age groups (Fig. 2A,B). The total number of germ cells containing at least one *sygl-1* ATS, which estimates the GSC number in the gonad, shows a moderate decline at Day 2 that progresses through Day 4, which is consistent with a previous report showing a reduction in SYGL-1 protein expression (Kocsisova et al., 2019) (Fig. 2A). Similarly, the total number of *sygl-1* ATS, which estimates the overall Notch transcriptional activation within the gonad, shows a progressive yet moderate decline through Day 4 (Fig. 2B). In addition, the average number of *sygl-1* ATS in each cell (Fig. 2C) and the fraction of GSCs containing >1 *sygl-1* ATS (Fig. 2D, colored bars), which estimate Notch activation at the cellular level, showed the GSCs with multiple *sygl-1* ATS progressively declined in aging, with the group of GSCs with 4 ATS almost completely depleted in Day 4 (Fig. 2C-D). However, aging did not affect expression of a Notch-independent gene, *let-858* (Fig. S2).

We then analyzed the individual *sygl-1* ATS intensities and the summed *sygl-1* ATS intensities that were pooled from all *sygl-1* chromosomal loci within each nucleus, which estimate the activity of Notch-dependent transcription at the *sygl-1* chromosomal level or the cellular level, respectively (Fig. 2E,F). Individual ATS intensities showed essentially no age-associated changes, whereas the summed ATS intensities showed a similar trend to that seen in the ATS numbers above (Fig. 2A,B; Fig. S1B,C). We also examined the nuclear size and the nucleolar size of the germ cells, which are closely related to longevity and cellular senescence (Son et al., 2019; Webster et al., 2009). We observed no changes in both organelles, indicating chronological aging does not immediately induce biological aging of the germ cells in early adulthood, at least until Day 4 (Fig. 2G,H; Fig. S3). We conclude that Notch-dependent transcription and the GSC pool are affected by aging all at molecular, cellular, and tissue levels as expected albeit to a moderate degree.

### Aging disrupts spatial regulation of Notch-dependent transcription in the germline and induces GSC dislocation

**Fig. 3.**
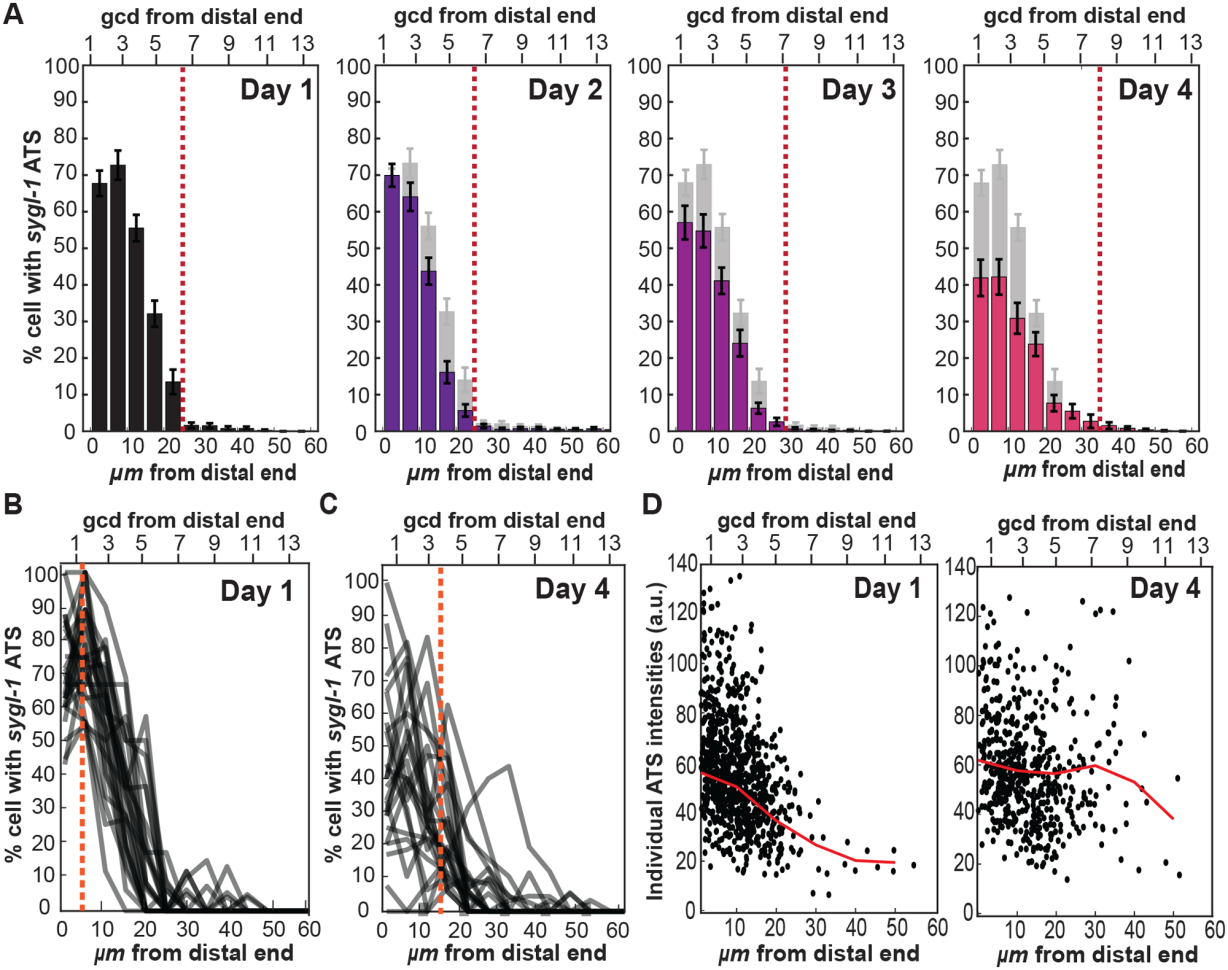
**Aging induces a progressive shift in the spatial pattern of Notch transcriptional activation.** (A) The percentage of cells containing at least one *sygl-1* ATS was plotted as a function of the distance from the distal end of the gonad for each age to estimate the probability of Notch-dependent transcriptional activation in each position relative to the distal end. The red dashed line marks the proximal boundary of the GSC pool, where the percentage is below 2.5%. *n*=27, 25, 26, and 24 gonads for Day 1, 2, 3, and 4 respectively. (B,C) Line plots of the percentage of cells containing at least one *sygl-1* ATS against the distance from the distal end of individual gonads overlaid at Day 1 (B) or Day 4 (C). (A) is the average of all these individual plots. The orange dashed line indicated the average peak of all the percentage of cells containing at least one *sygl-1* ATS. (D) The individual *sygl-1* ATS intensities are plotted against the distance from the distal end of Day 1 and 4. Each dot represents one *sygl-1* ATS. *n*=931 and 550 ATS for Day 1 and 4, respectively.

Notch-dependent transcription is steeply graded within the GSC pool under normal conditions, with its probability and activity highest next to the DTC/niche (Crittenden et al., 2019; Lee et al., 2016; Lynch et al., 2022). This graded spatial pattern of Notch activation is crucial for regulating the size and function of the GSC pool (Crittenden et al., 2019; Lee et al., 2016; Lynch et al., 2022). Maintaining the ‘normal’ gradient of Notch activation is key to tissue integrity, homeostasis, and functionality since its dysregulation can result in pathologies such as tumorigenesis or stem cell loss (Izumchenko et al., 2015; Kaylan et al., 2018; Lee et al., 2016; Mishra et al., 2001). We hypothesized that the spatial pattern of Notch-dependent transcription changes during aging, leading to germline depolarization and PZ shrinkage. To test the hypothesis, we recorded the percentage of the germ cells containing *sygl-1* ATS as a function of the distance from the distal end of the gonad, which estimates the probability of Notch transcriptional activation at various positions relative to the DTC/niche, from Days 1–4 (Fig. 3A). The Day 1 worms showed the ‘normal’ spatial pattern for *sygl-1* transcription as expected, exhibiting a steep gradient within the GSC pool (0–25 μm from the distal end), from about 70% at the distal-most region to less than 2.5% at the border of the GSC pool (Fig. 3A; Day 1, red dashed line) (Lee et al., 2016). The graded pattern was maintained through aging (Fig. 3A), but the percentage of *sygl-1* ATS-containing cells at the distal gonad steadily and significantly decreased as the worm aged, flattening the gradation (Fig. 3A; Fig. S4A; 0–15 μm from the distal end). Notably, the proximal pool of GSCs gradually increased during aging (Fig. 3A; 15–35 μm from the distal end), extending the GSC boundary more proximally (Fig. 3A; red dashed lines).

To understand how aging increased Notch activation in the proximal pool of the GSCs, we overlaid individual plots for Day 1 or Day 4 and examined the variability in the *sygl-1* transcriptional pattern within individual gonads (Fig. 3B,C), which revealed the details masked when averaging the spatial patterns together (Fig. 3A). The Day 1 worms showed most individual spatial patterns similar to the averaged pattern with small variability (compare Fig. 3A,B; Day 1). In most Day 1 gonads, the percentage of *sygl-1* ATS-containing cells peaked generally at ∼5 µm from the distal end of the gonad with a steep gradient that ends at around 25 μm (Fig. 3B; orange dashed line). In Day 4 gonads, however, the graded pattern became less obvious, and its average peak was shifted farther, ∼15 µm, from the distal end with relatively high variability in its position (Fig. 3C; orange dashed line). Over a third of Day 4 gonads completely lost *sygl-1* ATS-containing cells at the distal gonad and exhibited premature GSC differentiation at the distal gonad, containing cells in meiotic prophase at a significantly higher rate than Day 1 gonads (Fig. 3C and Fig. S4B,C). Consistently, the spatial pattern of *sygl-1* ATS intensities, which estimates the Notch activity with respect to the relative position from the distal end of the gonad (Fig. 3D), and *sygl-1* mRNAs, which is another Notch readout (Fig. S5A,B), also showed a similar locational shift toward the proximal region as the worm ages albeit to a lesser degree, when overlaying the individual spatial patterns of percent cells with *sygl-1* mRNA above basal level (Fig. S5C,D). We conclude that aging breaks the ‘normal’ spatial pattern of Notch-dependent transcription, shifting its peak to a proximal gonad, which in turn disrupts the germline tissue polarity.

### The DTC/niche nucleus also gradually drifts away from the distal end of the gonad during aging, moving the Notch-responsive GSC pool more proximally

**Fig. 4.**
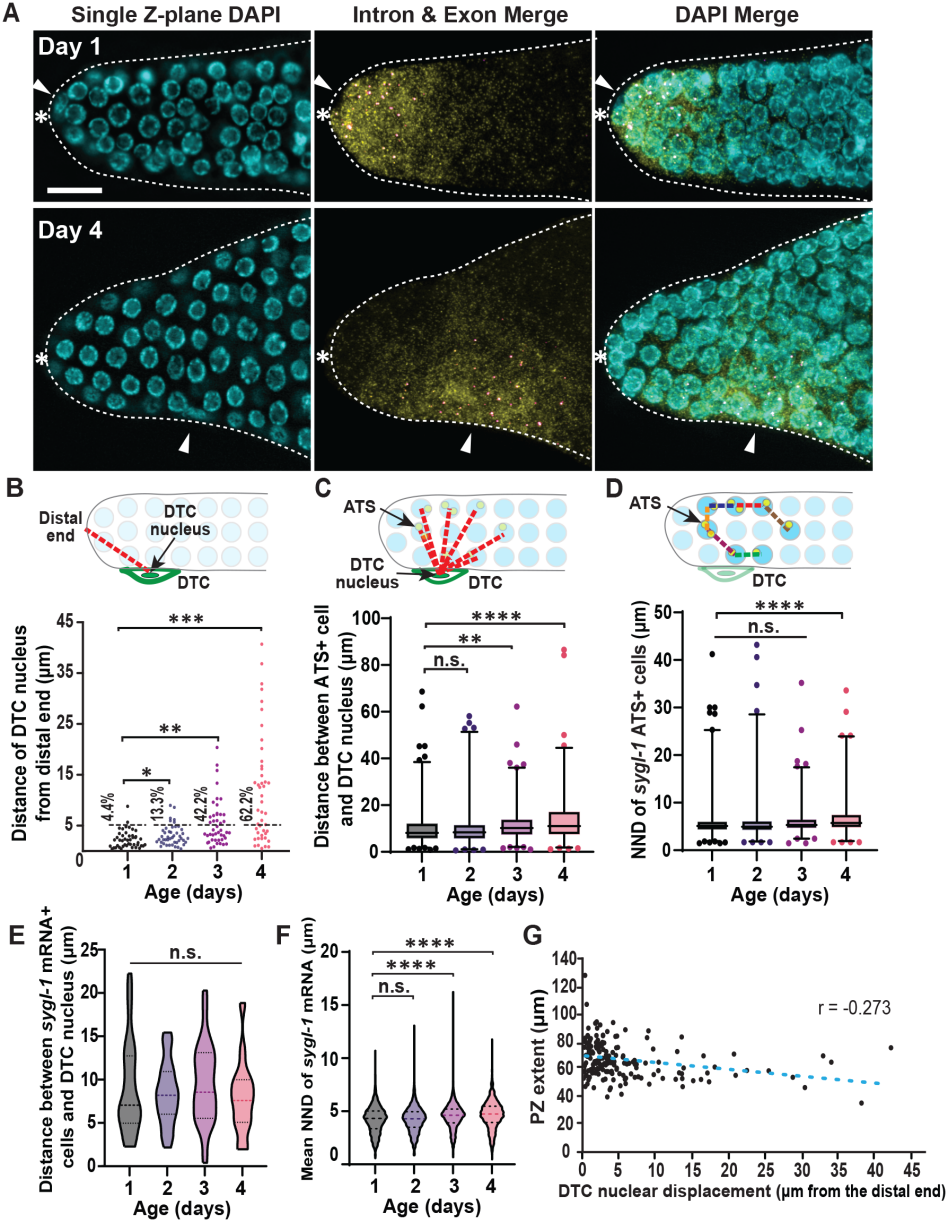
**Notch activation occurs near the DTC/niche nucleus regardless of the aging stage.** (A) Representative *sygl-1* smFISH images of Days 1 and 4. Left: single z-plane DAPI; middle: intron and exon merge; left: DAPI, intron and exon channel merge. White arrowhead indicates the DTC nuclear position. White asterisk indicates distal end of the gonad. (B) A diagram illustrating the Euclidean distance between the DTC/niche nucleus and the distal end of the gonad, indicating the amount of nuclear shift, are measured through aging. *n*=45 gonads for all ages. Error bar: the standard deviation. Black dashed line indicates the boundary where majority of the DTC nuclei reside in Day 1. Percentages listed are the number of gonads with DTC nuclear position that exceed the boundary (∼5 µm). (C) A diagram demonstrating the quantification of distance between the *sygl-1* ATS-containing cells and the DTC/niche nucleus, estimating the clustering of Notch activation to the nucleus through aging. For all box-and-whisker diagrams in this study, the middle line shows the median; top and bottom of box are the third and first quartiles, respectively; whiskers, maximum and minimum of data points; circles, outliers (value greater than 1.5× first or third quartile from the median). (D) The shortest distance between two neighboring cells that contain at least 1 *sygl-1* ATS (the nearest neighbor distance, NND) are shown for all ages. (A–C) *n*=621, 460, 508, and 444 ATS for Day 1, 2, 3, and 4 respectively. (E) The distance between the cells containing *sygl-1* mRNAs above the basal level and the DTC/niche nucleus. (F) The nearest neighbor distance (NND) of cells with *sygl-1* mRNAs above the basal level. (E,F) *n*=5462, 3913, 4856, and 5256 nuclei for Day 1, 2, 3, and 4 respectively. (G) A linear regression plot of the progenitor zone extent and the DTC nuclear shift from the distal end of the gonad for all ages. *n*=180 gonads.

What drives the locational shift of Notch activation during aging? Is the shift due to age-induced changes in the location, function, or structure of the DTC/niche, or is it merely stochastic? To address these questions, we examined the structural and physiological features of the aging DTC/niche (Fig. 4). The DTC/niche is a mononuclear mesenchymal cell with its nucleus typically located at the distal end of the gonad through development and at Day 1 (Kimble and White, 1981) (Fig. 4A; Fig. S6; Day 1). We first recorded the distance between the distal end of the gonad and the DTC/niche nucleus in the aging gonads, which revealed an age-associated progressive shift of the DTC/niche nucleus away from the distal end, from about 2 μm in average at Day 1 to about 12 μm at Day 4, over five times farther from the distal tip (Fig. 4B; individual replicate plots shown in Fig. S6B–D). While over 95% of Day 1 gonads have the DTC/niche nucleus within the first 5 μm of the gonad, only 38% of Day 4 retain it within 5 μm from the distal end of the gonad with its distance to the distal end as long as 42 μm (Fig. 4B). The age-associated shift of the DTC/niche, *sygl-1* ATS, and mRNAs also occurred in essentially the same manner in the mated worms, in which external sperm were constantly provided to keep fertilization during aging, suggesting that mating and egg production do not prevent the age-induced GSC shift (Fig. S6A).

Given the similar proximal shift of Notch activation (Fig. 3) and the DTC/niche nucleus (Fig. 4B), we postulate whether there is a link between them. For example, *sygl-1* ATS may appear only near the DTC/niche nucleus, keeping them nearby through aging. We first measured the distances between the DTC/niche nucleus and all *sygl-1* ATS-containing cells and compared them between all age groups (Fig. 4C). The average distance was about 10 μm at Day 1, which is smaller than half the length of the GSC pool (25 μm), and it only increased slightly through aging with 12 μm at Day 4, smaller than a one-germ-cell-diameter increase (Fig. 4C). In addition, the nearest neighbor distances (NNDs) of *sygl-1* ATS-containing cells in each gonad, which estimate how clustered the Notch-responsive GSCs are, showed little to no changes during aging, consistent with no age-induced changes in the GSC pool extent (Fig. 4D; Fig. S5E). These results indicate that Notch activation occurs near the DTC/niche nucleus and this proximity remains through aging. We also used *sygl-1* mRNAs instead of ATS for similar assays, which showed essentially the same results, further supporting the idea of the sustained proximity between Notch activation and the DTC/niche nucleus (Fig. 4E,F). Similarly, the size of the *sygl-1* ATS-rich region as well as mRNA-rich region remains unchanged during aging (Fig. S5E,F), despite its age-induced locational shift (Fig. 3; Fig. S5A,B). Notably, we find a moderate correlation between the distance the DTC/niche nucleus drifted and the PZ extent (Fig. 4G), suggesting that the age-associated shift of the DTC/niche nucleus and GSC dislocation at least in part induces the age-associated PZ shrinkage and offspring decline (Figs 1B,C and 4G).

### The distance to the DTC/niche nucleus determines Notch-dependent transcriptional activity in the GSC

**Fig. 5.**
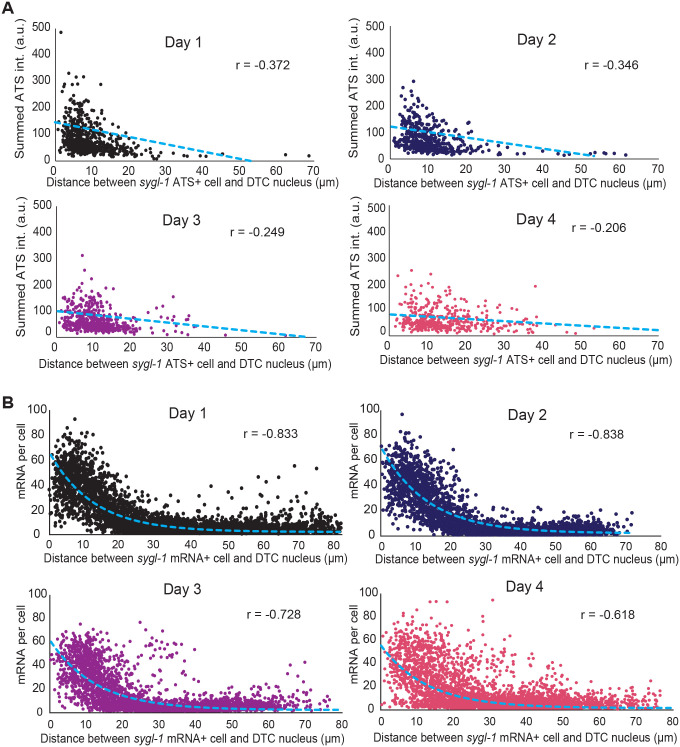
**Notch-dependent *sygl-1* transcriptional activity is correlated with the proximity to the DTC/niche nucleus.** (A) The summed *sygl-1* ATS intensities in each cell are plotted against the distance between the corresponding cell and the DTC/niche nucleus for all ages. r: Pearson's correlation coefficient. *n*=621, 620, 508, and 444 nuclei for Day 1, 2, 3, and 4 respectively. (B) Total number of mRNAs per cell as a function of the distance between the corresponding cell and the DTC/niche nucleus. *n*=5462, 3913, 3914, and 3914 cells for Day 1, 2, 3, and 4 respectively. (A,B) The blue dashed line indicates the line fitting model to calculate Pearson's r value.

To further investigate the link between the DTC/niche nucleus and Notch activation, we examined the summed *sygl-1* ATS intensities in the individual GSCs, estimating Notch transcriptional activity in each cell, with their positions relative to the DTC/niche nucleus (Fig. 5A; Fig. S7A). The analysis revealed that there is a modest correlation between the *sygl-1* transcriptional activity in the GSC and its proximity to the DTC/niche nucleus in Day 1. This correlation remained through aging, although the correlation became slightly weaker at older stages (Fig. 5A; Fig. S7A; Days 3 and 4). We also used another Notch readout, *sygl-1* mRNAs for a similar analysis, which showed similar but much stronger correlations throughout aging with a slight decrease in more aged groups similar to ATS analysis (Fig. 5A,B; Fig. S7B). The same analysis using the individual gonads showed the same correlations through aging, confirming the results (Fig. S7), suggesting that the proximity to the DTC/niche nucleus plays a critical role in determining how active Notch-dependent transcription will be.

### Analyses with a second Notch target, *lst-1*, confirms the age-induced progressive shift of the Notch-responsive GSC pool

**Fig. 6.**
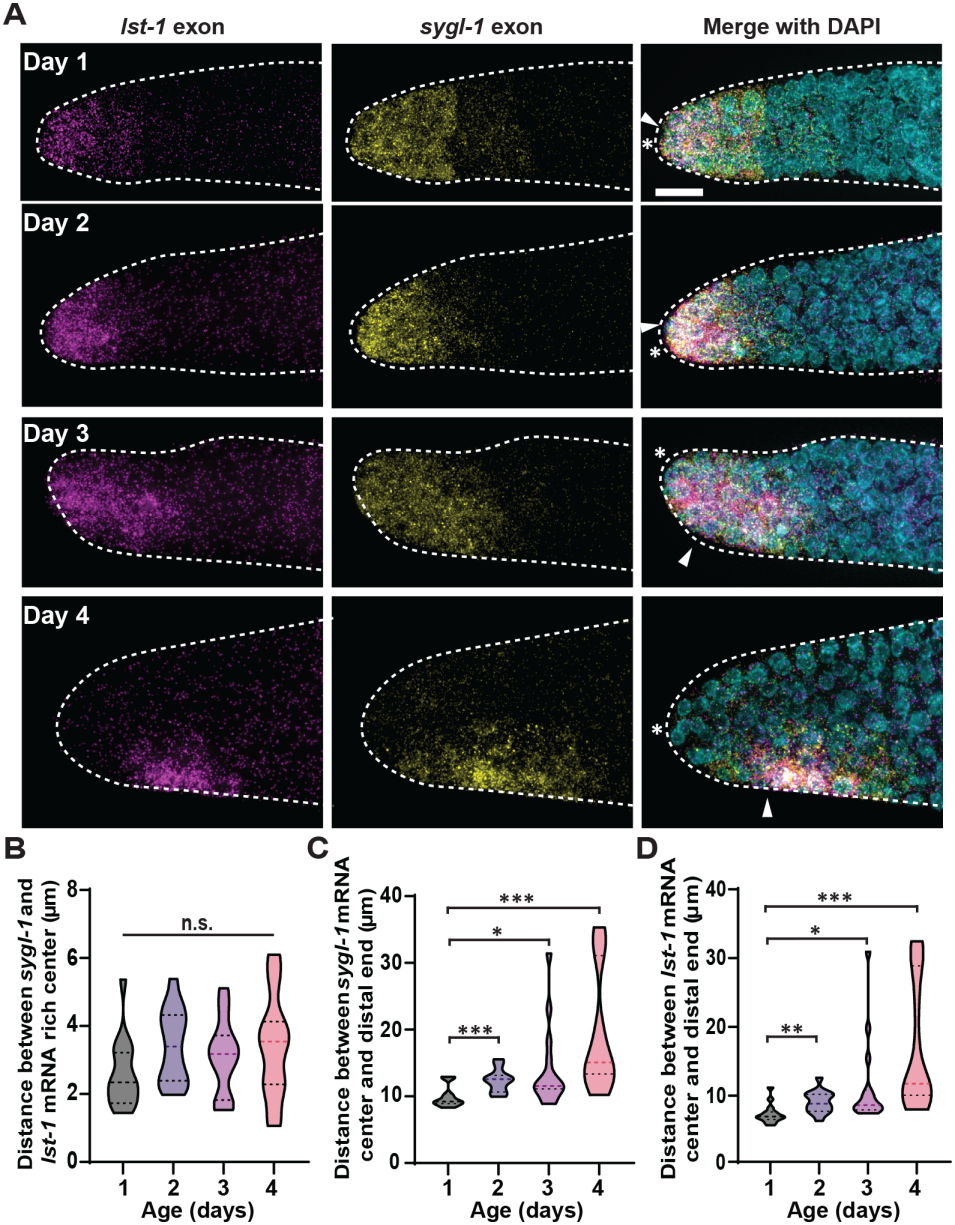
**Notch target gene, *lst-1*, exhibits an age-induced shift of cytoplasmic mRNAs.** (A) Representative of *lst-1* and *sygl-1* exon smFISH images from Day 1–4. Left: *lst-1* exon; middle: *sygl-1* exon; left: DAPI and *lst-1*/*sygl-1* exon channels merge. White asterisk indicates distal end of the goand. White arrowhead indicates DTC/niche nucleus. (B) The Euclidean distance between the center of *sygl-1* and *lst-1* mRNA regions. (C) The Euclidean distance between the center of *sygl-1* and the distal end of the gonad. (D) The Euclidean distance between the center of *lst-1* and the distal end of the gonad. (B–D) *n*=15 gonads for all ages.

To confirm our findings about the age-induced changes in the germline including the proximal shift of Notch activation, which was seen primarily with *sygl-1* transcripts, we used a second Notch target, *lst-1*, a well-characterized gene encoding another stem cell effector critical to GSC maintenance (Kershner et al., 2014; Kocsisova et al., 2019; Shin et al., 2017). Our *lst-1* smFISH using the *lst-1* exon-specific probe set revealed that *lst-1* mRNA-expressing cells also shift to a more proximal region in an age-dependent manner (Fig. 6A), consistent with the results with *sygl-1* (Fig. 1D). A simultaneous visualization of *sygl-1* and *lst-1* mRNAs showed that the majority of the *lst-1*-expressing cells, if not all, also contain *sygl-1* mRNAs, and this co-existence persists through aging at least to Day 4 with the *sygl-1* and *lst-1* mRNA-rich regions stays very close (Fig. 6A,B). In addition, both the *sygl-1* and *lst-1* mRNA-positive germ cell pool, which is essentially the Notch-responsive GSC pool, progressively drifted away from the distal end of the gonad during aging along with the DTC/niche nucleus to essentially the same degree (Fig. 6A–D), confirming our findings above only with *sygl-1* (Figs 3–5).

### The DTC/niche experiences age-induced changes in its structure, morphology, and integrity even in early adulthood

**Fig. 7.**
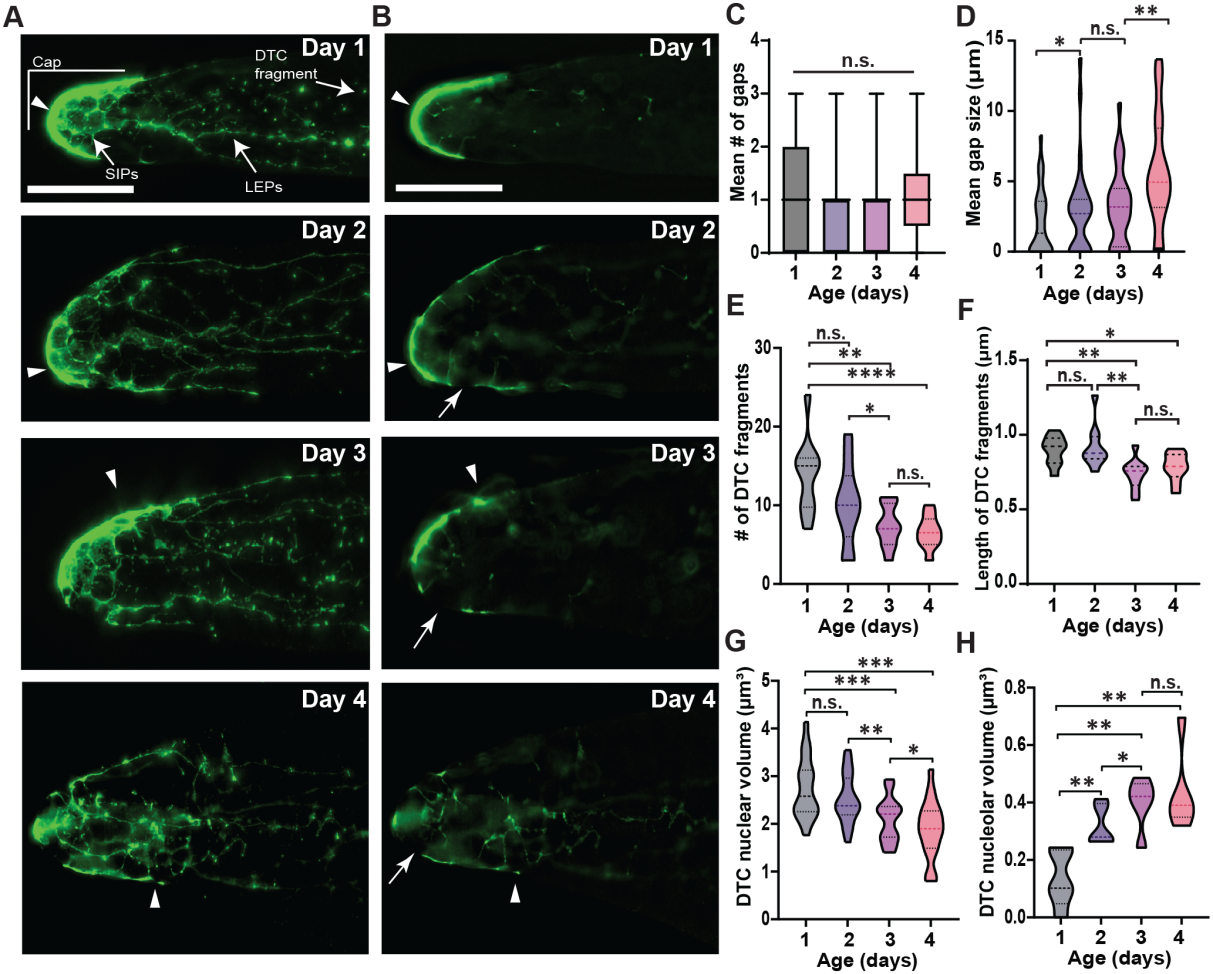
**Aging induces structural and morphological changes of the DTC/niche.** (A) Myristoylated-GFP expressed under a DTC-specific promoter, *lag-2*, visualizes the plasma membrane of the DTC/niche. The maximum Z projection is shown. LEPs: long external processes. SIPs: short intercalating processes. (B) A single Z plane of the myr-GFP image shown in Fig. 6A for each age. Arrow: DTC/niche gap. (A,B) Arrowhead: DTC/niche nucleus. (A,B) Scale bar: 20 μm. (C) The mean number of gaps in the DTC cap for all ages. (D) The size of the DTC gap, a region missing its plasma membrane for all ages. *n*=30, 30, 30, and 29 gonads for Day 1, 2, 3, and 4 respectively. (E) The number of detached DTC fragments, isolated small DTC/niche membranes. (F) The length of the detached DTC fragments. (E,F) *n*=10 gonads for all ages. (G) DTC nuclear volume was measured for all ages to estimate the changes in nuclear size. *n*=20 nuclei for each aging stage. (H) DTC nucleolar volume in all ages. *n*=5 nucleoli for each aging stage.

The *C. elegans* DTC/niche has an extensive structure, consisting of a cap that encapsulates a distal half of the GSC pool and cellular processes that extend from the cap and reach the germ cells even beyond the GSC pool (Byrd et al., 2014; Crittenden et al., 2006; Wong et al., 2013). The processes fall into two groups, the long external processes (LEPs) that are typically thick and long to reach the end of the progenitor zone (PZ), and the short intercalating processes (SIPs) that infiltrate into the gonad to physically interact with the germ cells mostly at the distal gonad (Byrd and Kimble, 2009; Byrd et al., 2014). Visualization of the DTC/niche plasma membrane using the myristoylated-GFP (myr-GFP) expressed under a DTC/niche specific promoter, *lag-2*, revealed the cellular structure of the DTC/niche, including the cap, LEPs, and SIPs, through aging (Fig. 7A).

The length of LEPs increased during aging, with LEPs in Day 4 about 30% longer than Day 1 as previously reported (Crittenden et al., 2006) (Fig. S8A). However, there were little to no changes in the SIP extent from Days 1–4 (Fig. S8B), suggesting essentially no change in connectivity between the DTC/niche and the GSC pool during aging at least until Day 4. The cap of the DTC/niche has a few gaps, where the myr-GFP signal is missing, and therefore the DTC/niche cannot make physical contact with the germ cells for Notch activation (Byrd and Kimble, 2009; Byrd et al., 2014; Crittenden et al., 2006; Lee et al., 2016) (Fig. 7B; white arrows).

Similar to the SIP extent that does not change, the total number of DTC/niche gaps did not change during aging, indicating that aging does not induce new gap formation (Fig. 7C and Fig. S8B). However, the DTC/niche gaps generally grew larger during aging, over twofold from Days 1–4, indicating that aging may disrupt the cellular integrity of the DTC/niche (Fig. 7D). The number of cellular fragments detached from the DTC/niche decreased significantly from Days 1–4 (Fig. 7E), suggesting that aging may affect the DTC/niche dynamics in forming new processes or severing newly formed processes, which occurs frequently at Day 1 under normal conditions (Wong et al., 2013). The size of the detached DTC fragments, however, did not show noticeable age-induced changes (Fig. 7F). We compared the nuclear size and the nucleolar size of the DTC/niche between age groups, which showed a progressive decrease and an increase during aging, respectively (Fig. 7G-H). Both changes suggest the senescence of the DTC/niche or a decreased capacity for DTC/niche longevity early in adulthood (Son et al., 2019), consistent with the age-associated decline in DTC/niche integrity (Fig. 7G,H). Altogether, these results show that the DTC/niche undergoes structural and morphological changes during aging and that these age-induced changes coincide with the dislocation of the DTC/niche nucleus, which disrupts the spatiotemporal regulation of Notch activation and germline tissue polarity, leading to a decline in the mitotic germ cell pool and fecundity.

## DISCUSSION

This work analyzed the stem cell niche aging process and its consequences *in vivo* using the aging *C. elegans* gonad with a focus on the DTC/niche-GSC interactions that are mediated by Notch signaling. We assessed the age-induced changes in Notch activation at the molecular, cellular, and tissue levels by examining Notch-dependent transcriptional activation and its activity through aging (Lee et al., 2016, 2017; Lynch et al., 2022). This study particularly focuses on aging in early adulthood, from the young adult stage (Day 1; 24 h post L4), where the hermaphrodite worm becomes gravid, to Day 4 since L4, when the worm concludes self-fertilization and ceases embryogenesis without the external sperm from mating (Scharf et al., 2021; Van Bael et al., 2018). We show that both the germline tissue and the DTC/niche experience biological aging even in early adulthood, Days 1–4, exhibiting a progressive locational shift of the GSC pool and a disruption of the germline tissue polarity, while Notch-dependent transcriptional activation in the GSCs are affected only moderately. We reveal that these changes are due to an age-induced shift of the DTC/niche nucleus with progressive changes in the structure and morphology of the DTC/niche. Also, we find a strong correlation between Notch transcriptional activity and its proximity to the DTC/niche nucleus. Notably, continued gametogenesis by mating did not prevent these aging consequences, exhibiting age-induced changes and declines as unmated worms did. Below we place the insights gained in context and discuss their broader implications.

### Germline tissue aging is induced by the aging of the DTC/niche and occurs at all biological levels including molecular and cellular levels but each at a different rate

One of the unexpected findings in this study is that Notch-dependent transcriptional activation and its activity in the *C. elegans* germline, which is crucial for regulating stemness of the GSCs, reduce only moderately during aging, at least until 3 days after Day 1 (Day 4), although the germline functions, including PZ maintenance, germline polarization, and fecundity, drastically decline during the time (Figs 1B,C, 2, 3; Fig. S4). Although the age-induced tissue-level declines have consistently been documented (Crittenden et al., 2006; Kocsisova et al., 2019; Scharf et al., 2021), germline tissue aging has not been analyzed at molecular, chromosomal, and cellular levels with direct, high-resolution transcriptional reporters for Notch activation and GSC identification until this study. The size of the nucleus and the size of the nucleolus of the germ cells, both of which have been established as biomarkers to indicate longevity of the cells and therefore estimating their cellular senescence status, did not change during aging at least until Day 4 (Fig. 2G–H). In addition, transcription of a Notch-independent gene, *let-858*, is unchanged during the time (Fig. S2), suggesting that the aging effects on the GSCs and their cellular senescence are minimal, consistent with the recent findings that the stem cells ages minimally under normal conditions (Kalamakis et al., 2019; Morrow and Moore, 2019; Navarro Negredo et al., 2020; Oh et al., 2014). Interestingly, the nuclear size and the nucleolar size of the DTC/niche gradually decreased or increased, respectively, both indicating the diminished longevity during aging from Days 1–4, which occur with structural and morphological changes of the DTC/niche such as an increase of the gap size and the decrease of the DTC fragment count (Fig. 7). These results suggest that the DTC/niche, a somatic tissue, may experience biological aging much quicker than GSCs and that a germline tissue decline may be mainly associated with the cellular aging of the DTC/niche rather than GSC senescence *per se*. DTC/niche aging occurs early in adulthood similar to the aging of some human tissues that begins soon after puberty, such as connective tissue of the eye and skin (Freemont and Hoyland, 2007; Pardue and Sivak, 2000), thus a similar niche aging and functional decline we observed in the *C. elegans* gonad may occur in humans as well. In general, older gonads exhibit higher variability in Notch-dependent transcription, suggesting that the age-induced declines may be in part due to the regulation of DTC/niche becoming weak and inconsistent or GSCs becoming more susceptible to gene expression noise, which possibly leads to inefficient GSC self-renewal and early differentiation. Analyzing the aging process and its effects in other tissues or organisms and expanding the assays to even older stages will broaden our current understanding of stem cell and niche aging.

### The DTC/niche nucleus defines the Notch-responsive GSC pool

We observed a progressive age-induced change in the Notch activation pattern in the GSC pool, from a steep probability gradient at Day 1 to a more flattened gradient at older stages both in *sygl-1* ATS occurrence and mRNA abundance (Fig. 3; Fig. S5). In the aged groups, the probability of Notch activation at the distal-most gonad, where it is normally highest at Day 1, significantly decreases whereas it increases at the border of the GSC pool, extending the pool more proximally (Fig. 3A–C), which seemingly contradicts the slight reduction of Notch activation in the aging gonads (Fig. 2A–C). However, we find that these changes are due to the locational shift of Notch activation in the gonad, which causes a mislocation of the GSC pool, losing Notch-responsive GSCs at the distal gonad but gaining misplaced GSCs at a more proximal region (Fig. 3A–C). Examining a second Notch target, *lst-1* further confirmed the conclusions (Fig. 6). A previous study reported a slightly smaller number of DTC/niche nucleus shift occurrence in an aged gonads (∼15% within 15 gcd versus our 27% within 15 gcd; see Fig. 4B) (Kocsisova et al., 2019). The slight discrepancy may stem from different mating conditions, with which the previous study used mated aging worms while we use unmated hermaphrodites for most analyses. Regardless, both studies overall show consistent results in decreased Notch activation and Notch target expression during germline tissue aging. Then, what drives this locational shift of Notch activation? An anti-correlation between Notch-dependent transcriptional activation and its distance to the DTC/niche nucleus, which persists through aging albeit a slight decrease in the older gonads (Fig. 5A), indicates that Notch activation does not occur uniformly within the GSC pool but does more strongly near the DTC/niche nucleus. The correlation is even stronger when considering *sygl-1* mRNA abundance instead of the ATS (Fig. 5B), which is possibly due to the difference in half-lives of *sygl-1* ATS and mRNAs, which have been estimated as <15 mins and >1 h, respectively (Lee et al., 2016). The correlation between the ATS and its proximity to the DTC/niche nucleus could be underestimated as roughly half the GSCs do not exhibit *sygl-1* ATS at any given time due to the stochastic and sporadic nature of *sygl-1* transcription (Lee et al., 2016, 2019). In contrast, the mRNAs retain much longer time and therefore seen at any given time, which can be more reflective of transcriptional activation over a longer period, showing the correlation more clearly. Because the DTC/niche nucleus drifts away from the distal end of the gonad during aging, the zone of Notch activation also moves along with the DTC/niche nucleus, causing the age-induced shift of the Notch-responsive GSC pool (Fig. 4). Consistently, a similar locational shift of the DTC/niche nucleus and SYGL-1 protein expression pattern have been observed in a previous study (Kocsisova et al., 2019).

The strong correlation between Notch activation and the proximity of the DTC/niche nucleus (Fig. 5; Fig. S7) implies that most transcripts or proteins of Notch ligands do not travel far but stay close to the nucleus, or that the ligands are more prone to be degraded when they move away from the nucleus. Our results support that the DTC/niche nucleus determines the location of Notch activation in the germline and therefore defines the GSC pool. Also, our results reveal a moderate correlation between the DTC/niche nucleus shift and PZ extent (Fig. 4G), suggesting that the germline tissue polarity established by the ‘normal’ gradient of Notch activation is important for germline function and fecundity. The DTC/niche nucleus is typically located at the distal end of the gonad throughout *C. elegans* development, when the DTC/niche serves as a migratory cell, promoting the gonad migration and the germline tissue polarization (Byrd et al., 2014; Kimble and White, 1981). The shift of the DTC/niche nucleus coincides with the DTC/niche losing its leader function (Byrd et al., 2014; Cecchetelli and Cram, 2017), suggesting that the cessation of migratory cues may be involved in the initiation of the DTC/niche aging process.

### The link between the structure and morphology of the DTC/niche and its aging process

In addition to a shift of the DTC/niche nucleus, the DTC/niche undergoes structural and morphological changes during aging (Fig. 7). The long-extended processes (LEPs) grow longer during aging, which was also reported previously (Crittenden et al., 2006) (Fig. S8A). The size of the gaps, discontinuous regions in the DTC/niche cap, increases with aging while the number of gaps remains unchanged (Fig. 7C,D), indicating that aging itself does not create more gaps in the DTC/niche but the existing gaps become larger, possibly by the DTC/niche being stretched due to the extending SIPs and LEPs. The larger gaps in the aged DTC/niche may hamper the interactions between the DTC/niche and the GSCs, possibly contributing to a slight decline in the probability of Notch activation. These structural and morphological changes coincide with a decline both in the clustering of *sygl-1* ATS-containing cells to the DTC/niche nucleus (Fig. 4C) and the correlation between Notch activation and the proximity of the DTC/niche nucleus (Fig. 5) in the older gonads. Notably, the number of the DTC/niche fragments, small fragments detached from the main DTC/niche body, progressively decreases during aging (Fig. 7E). The small fragments were suggested to be the result of new branch formation attempts (Wong et al., 2013), thus a reduction of fragments may indicate a reduced DTC/niche dynamics to form new physical interactions with the GSCs. Fewer interactions between the DTC/niche and GSCs mean less GSC number and activity. The DTC/niche dynamics can play an important role in the aging process, which needs to be tested by timelapse imaging.

## MATERIALS AND METHODS

### Nematode maintenance

All strains were maintained at 20°C as described in (Brenner, 1974). The wild type was N2 Bristol. The transgene was as follows: LGIII: qSi153[Plag-2::myr-GFP; Pttx-3::DsRED] III (Byrd et al., 2014), where the strain was derived). For smFISH and immunofluorescence of the wild type and transgenic stains, animals were grown at 20°C until plates contained adult worms and eggs were present on the agar plate. The animals were synchronized using the hypochlorite treatment or the bleaching technique as described in (Porta-de-la-Riva et al., 2012) to produce synchronized L1 larvae. The L1 larvae continue to grow at 20°C until 24 h post-mid-L4 stage (young adult, Day 1). For aged worms (Days 2–4), worms were transferred to a new plate every 24 h until 4 days post-mid-L4.

### Strains used in this study

N2: wild type

JK4533: *qSi153[Plag-2::myr-GFP; Pttx-3::DsRED] III*

### Antibodies

Anti-Green Fluorescent Protein Mouse IgG 2A (ThermoFisher Scientific, A11120)

Anti-DAO-5 Mouse mIgG2B (AB 10573805)

IgG (H+L) Highly Cross-Adsorbed Donkey anti-Mouse, Alexa Fluor Plus 488, Invitrogen (ThermoFisher Scientific, PIA32766)

### Single molecule RNA fluorescent *in situ* hybridization and immunofluorescence (Co smFISH-IF)

Benchtop, gloves, and pipettors were wiped down with RNaseZap and RNase-free filtered tips and tubes were prepared. Synchronized plates were washed with 2 mL of non-RNase free 1× phosphate buffered saline solution with 0.1% Tween-20 (PBST) and transferred to a 60 mm plastic Petri dish cover. Then an additional 2–3 mL of PBST was added to the dish cover. To extrude the gonads, 0.25 M levamisole stock was first added in a 1:1000 dilution to the PBST, attaining a final levamisole concentration of 0.25 mM. Then using a sterilized scalpel, the worms were dissected behind the pharynx or before the rectum to release the gonads. Dissections were completed within 20 min to prevent any gonad deformation. Dissected worms were collected in a 1.5 mL RNase-free Eppendorf tube and spun down for 60 secs at 2000 rpm. The supernatant was removed, and the sample was fixed with PBST and 3.7% formaldehyde and incubated on a rotator at room temperature for 30 mins. The sample was then spun down to remove the supernatant into an organic waste bottle. To permeabilize the sample, 1 mL of RNase-free 1× PBS+0.1% Triton X-100 was added to the sample and incubated on a rotator for 10 min at room temperature. The sample was spun down at 2000 rpm for 60 s and the supernatant was removed. The sample was washed twice with 1 mL RNase-free PBST. The sample was spun down at 2000 rpm for 60 s and was inverted 5–6 times between each wash. The sample was spun down after the second wash and resuspended in 1 mL of RNase-free 70% ethanol. Samples were placed at 4°C overnight or up to 1 week.

To prepare the probe for the hybridization step, the dried probe mix (5 nmol) to 40 µL of RNase-free TE buffer (10 mM Tris-HCl, 1 mM EDTA, pH 8.0) to create a probe stock of 125 µM. The probe was then diluted to 1:20 (6.25 µM). Fixed samples were spun down and ethanol was removed. Samples were then equilibrated in smFISH wash buffer (2× SSC, 10% deionized formamide in nuclease-free water) for 5 min. While the samples equilibrated in the wash buffer, 48 µL of hybridization buffer (HB; Contains 1 g dextran sulfate, 1 mL 20× SSC, 7.3 mL H20 (or up to 10 mL volume), and 1 mL formamide is thawed and added to a new 1.5 mL RNase-free tube with 1 µL of each probe dilution (1:20; intron and exon of *sygl-1*; intron probe for *let-858*). The samples were then spun down for 60 s at 2000 rpm and the supernatants were discarded. The probe-HB mix was then added to the sample and incubated at 37°C for 4–72 h.

After hybridization, 1 mL wash buffer was added, spun down, and removed. To prepare the sample for immunofluorescence, 100 µL of blocking solution (PBST+0.5% BSA) was added and incubated for 30 min at room temperature. The blocking solution was spun down and removed. The primary antibody was diluted 1:200 in blocking solution and incubated overnight at 4°C. Samples were washed twice with 200 µL of blocking solution. The secondary antibody was diluted 1:1000 in blocking solution and DAPI was added at a concentration of 0.5 µg/mL. The sample was incubated in secondary antibody/DAPI at room temperature for 1–2 h. The sample was washed twice with 200 µL blocking solution, spun down, and removed. The samples were then resuspended in 10 µL of ProLong Gold mounting media and mounted on a glass slide in a dropwise manner. Slides are cured for 24–60 h and sealed.

### Identification of germ cells and localization of the distal tip cell (DTC/niche) nucleus

DAPI staining was used to assess cell row counts and progression into gametogenesis (mitotic or meiotic cells). Mitotic cells are identified by their round ‘donut-like’ shape due to the lack of DAPI expression in the center from a large nucleolus. Meiotic cells are presented in two manners, crescent-shaped transitionary cells, or as larger rounded cells with visible chromatin condensation in the center (Hillers et al., 2017). The DTC/niche nucleus position was identified using the DAPI staining. Key morphological differences between the DTC/niche nucleus and the GSCs include a lack of a dark center and an elliptical shape, allowing it to be easily distinguished from the rounded germ cell nuclei (Hillers et al., 2017). For measuring the DTC/niche nuclear shift, the Euclidean distance was measured from the most distal point of the gonad to the center of the DTC/niche nucleus.

### Progenitor zone extent measurement

The progenitor zone extent was assessed by measuring from the most distal end of the gonad to the end of the progenitor zone using a segmented line on ImageJ to consider the natural curve of the gonad. The boundary of the progenitor zone was determined by the presence of more than one cell presenting a crescent-shaped morphology (Byrd et al., 2014; Crittenden et al., 2006; Fausett et al., 2023; Gordon, 2020; Roy et al., 2016).

### *sygl-1/lst-1* exon overlay analysis

The center of the *sygl-1/lst-1* mRNA rich region was examined by creating an ROI of the mRNA rich region (>10 mRNA per cell) to identify the center. The Euclidean distance between *sygl-1/lst-1* centers was measured using the X, Y coordinates. The shift of *sygl-1/lst-1* mRNAs was measuring using a straight line on ImageJ from the center of the mRNA rich region to the distal end of the gonad.

### DTC processes and cap structure assessment

DTC/niche processes were measured in fixed germlines from the strain JK4533 containing *qSi153*(*Plag-2::myr-GFP*) stained with anti-GFP. The extent of long external processes (LEPs) was measured from the most distal end of the DTC/niche cap to the most proximal point of a continuous process. The extent of the short intercalating processes (SIPs) was measured from the most distal end of the DTC/niche cap to the most proximal point of the web-like processes. Structural changes to the cap such as gaps were measured using the segmented line function in ImageJ to measure the length of regions of the DTC cap lacking GFP expression as gaps as well as the number of gap occurrences. Detached DTC fragments were identified as GFP blobs >0.5 µm and are not connected to a process. Due to its amorphous shape, the fragment lengths were measured longwise using a straight-line tool on ImageJ.

### Widefield microscopy setup with THUNDER processing and image acquisition

Gonads were imaged using a Leica DMi8 (widefield microscope) equipped with a Leica HC PL APO 63x/1.40-0.60 NA oil immersion objective, LED8 fluorescence illuminator, and THUNDER Imager with exceptional computational clearing methods to remove excessive background. All gonads were imaged completely (depth >15 µm) with a Z-step size of 0.3 µm using the Leica Application Suite X (LAS X) acquisition software (Leica Microsystems Inc., Buffalo Grove, IL, USA). All imaging was done with LED8 light sources. Channels were sequentially imaged in decreasing wavelengths to avoid bleed-through and prevent any photobleaching from occurring.

The *sygl-1* exon probe (TAMRA) was excited at 555 nm (40%), and the signal was acquired at 540–640 nm (gain was set to high well capacity) with an exposure time of 200 ms. The *sygl-1* intron probe (Quasar 670) was excited at 635 nm (40%) and the signal was acquired at 625–775 nm (gain was set to high well capacity) with an exposure time of 250 ms. The *lst-1* exon probe (Quasar 670) was excited at 635 nm (40%) and the signal was acquired at 625–775 nm (gain was set to high well capacity) with an exposure time of 200 ms. The *let-858* intron probe (TAMRA) was excited at 555 nm (40% illumination), and the signal was acquired at 540–640 nm (gain was set to high well capacity) with an exposure time of 250 ms. GFP was excited at 475 nm (10% illumination), and the signal was acquired at 470–550 nm (gain was set to high well capacity) with an exposure time of 200 ms. DAPI was excited at 390 nm (10% illumination), and the signal was acquired at 400–480 nm (gain high well capacity) with an exposure time of 50 ms.

### Image processing using the custom-made MATLAB codes

All processes were implemented and automated using modified MATLAB (v.2.0) codes similar to the source code developed in our previous work (Crittenden et al., 2019; Lee et al., 2016; Lynch et al., 2022) with certain modifications to optimize the source code for use with widefield microscopy images. MATLAB R2021A with the ‘image processing’ toolbox was used for all image processing and analyses. The images acquired in this study were packaged in LIF format and were prepared for MATLAB processing: (1) rotate the image to orient the distal end to the left-hand side; (2) crop to the 54–65 µm mark from the distal end using ImageJ (3) and saving as TIFF format with their metadata (e.g. pixel size, z-step size, and the total number of z slices) intact.

The TIFF files of the images were then read into the MATLAB detection code and the X, Y, and Z coordinates and signal intensity of gonadal boundaries, nuclei, mRNAs, and ATS (nuclear spots) were recorded. Once the channels are separated and recorded, the gonadal boundary is set and determined using an RNA channel. The MATLAB function ‘imfindcircles’ was used to define each nuclear boundary by drawing concentric nuclear circles. The DAPI signal in each Z-plane was then normalized to the mean background intensity within the same Z-plane, where there were no nuclei present. These concentric circles in different Z-planes were fit together in the process of creating a three-dimensional (3D) spherical nucleus by drawing a best-fit sphere and were scored as a part of the nuclei based on two criteria: (1) concentric circles were detected in ≥4 consecutive Z-planes and (2) the variation in Euclidean distances (X, Y direction) of the centers within the concentric circles was <0.5 µm. To estimate the nuclear size and DAPI intensity, the radius of the 3D spherical nucleus was recorded and the DAPI signal within the 3D spherical nucleus was summed and recorded, respectively.

For RNA detection, it was divided up into two categories: ATS detection and mRNA detection. For the detection of ATS, a Gaussian local peak detection method (‘FastPeakFind’ function) (Natan, 2013) and a signal-to-local background ratio method were used independently to detect ATS using the channel obtained from the intron-specific probe. Each potential ATS candidate within a Z-plane were detected based on the following criteria for *sygl-1*: (1) the signal-to-mean overall background ratio is greater than 1.0 in the germline; (2) the signal to local background (the area located within 3× the distance from the ATS center) ratio is greater than 1.05. These ATS candidates were then overlaid with the MATLAB-generated nuclei and filtered out for true ATS using the following criteria: (1) RNA spots in the intron-specific smFISH channel were localized to a nucleus; (2) the intron probe nuclear RNA spot is co-localized with its corresponding exon-specific probe nuclear spot; and (3) the signal intensities from both exon and intron probes were at least as bright as a single mRNA spot that is detected.

For the detection of mRNA, the MATLAB code was set up in a similar manner as the ATS detection described above with the exception that it was based on *sygl-1* exon-specific probes, with modification applied. The DetectRNAexon function was modified to improve detection of mRNAs in close proximity as reported previously (Crittenden et al., 2019; Lynch et al., 2022). Through new modifications, mRNAs are detected directly through the exon signal, rather than calculating exon signal through the intron channel The ATS intensities measured from the exon probes were also used in the direct comparison between ATS and mRNAs intensities and numbers. The mean of cytoplasmic mRNA intensities for each 3D gonad was set to 1 arbitrary unit (a.u.), and the germ cell boundary was determined using a 3D Voronoi diagram to estimate the number of mRNAs per germ cell (Ledoux, 2007; Yan et al., 2013). This allows the reconstruction of the germ cell boundary at the midpoint between two neighboring nuclei. The size or radius of each germ cell was restricted to 3 µm from the nucleus center.

After the analysis was completed, MATLAB and GraphPad Prism were used to conduct statistical tests and visualize data where the dataset underwent normality tests (Anderson–Darling normality test). If the data set met the requirements for parametric statistical analysis, ANOVA and *t*-tests were performed. If the data set did not satisfy the requirements for parametric analysis, the Kolmogorov–Smirnov (KS) test (a nonparametric version of the *t*-test) was used to compare data.

## Supplementary Material

10.1242/biolopen.060261_sup1 Supplementary information
