# Supplementary material for: Aging disrupts spatiotemporal regulation of germline stem cells and niche integrity
Source: Biol Open. 2024 Jan 15;13(1):bio060261. doi: 10.1242/bio.060261 (PMC10810562; doi:10.1242/bio.060261)
Supplement: Supplementary information [file biolopen-13-060261-s1.pdf]

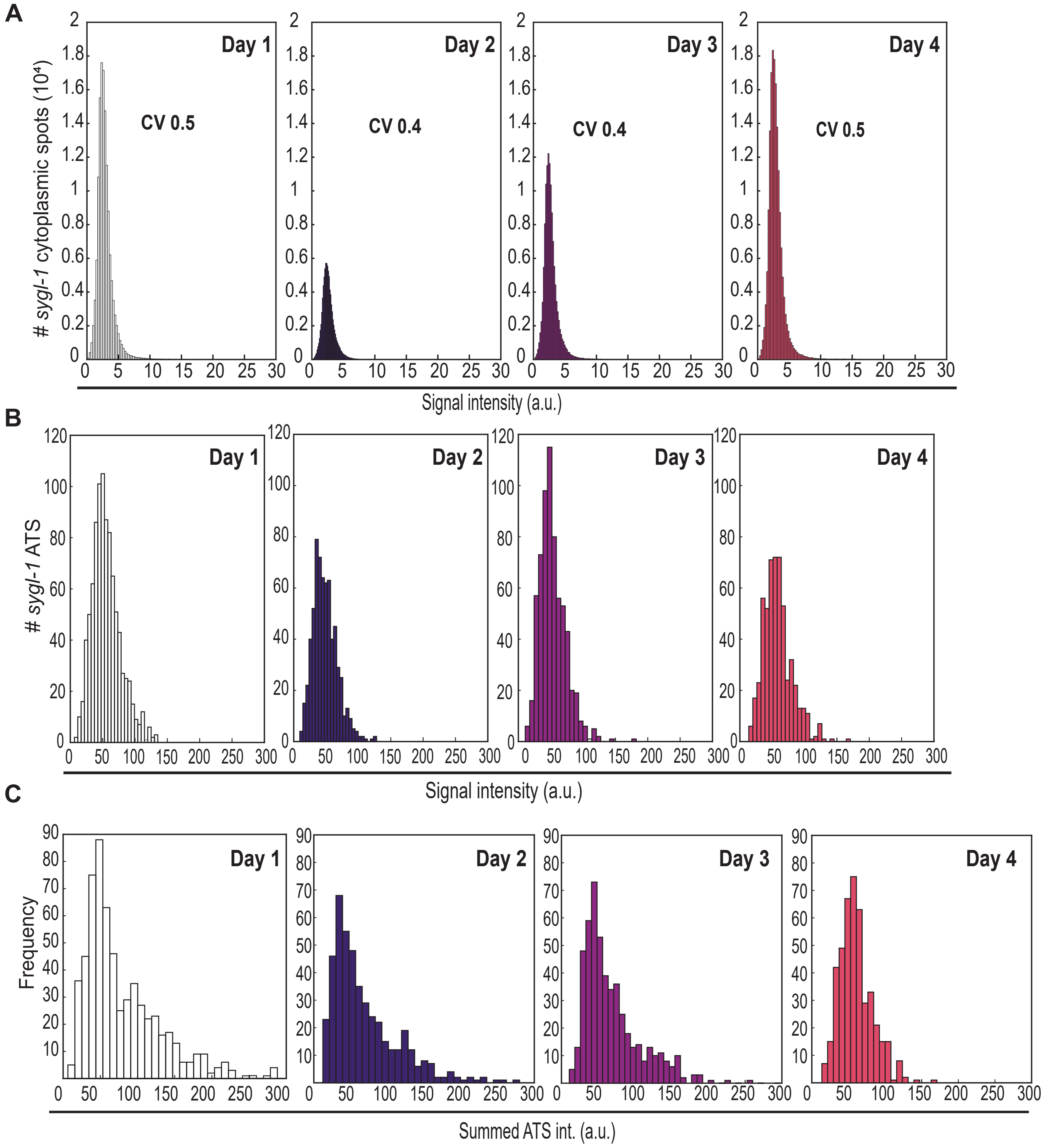

Fig. S1. (A) Histogram of cytoplasmic *sygl-1* mRNA signal intensities. Coefficient of Variance (CV) values listed on the Graph. n=135,914 mRNA for Day 1; 88,297 for Day 2; 124,661 for Day 3, and 168,138 for Day 4. (B) Histogram of *sygl-1* ATS signal intensities. n=931, 661, 660, and 550 ATS for Day 1, 2, 3, and 4 respectively. (C) The histogram of the summed *sygl-1* ATS intensities. n=621, 460, 508, and 444 nuclei for Day 1, 2, 3, and 4 respectively.

Biology Open • Supplementary information

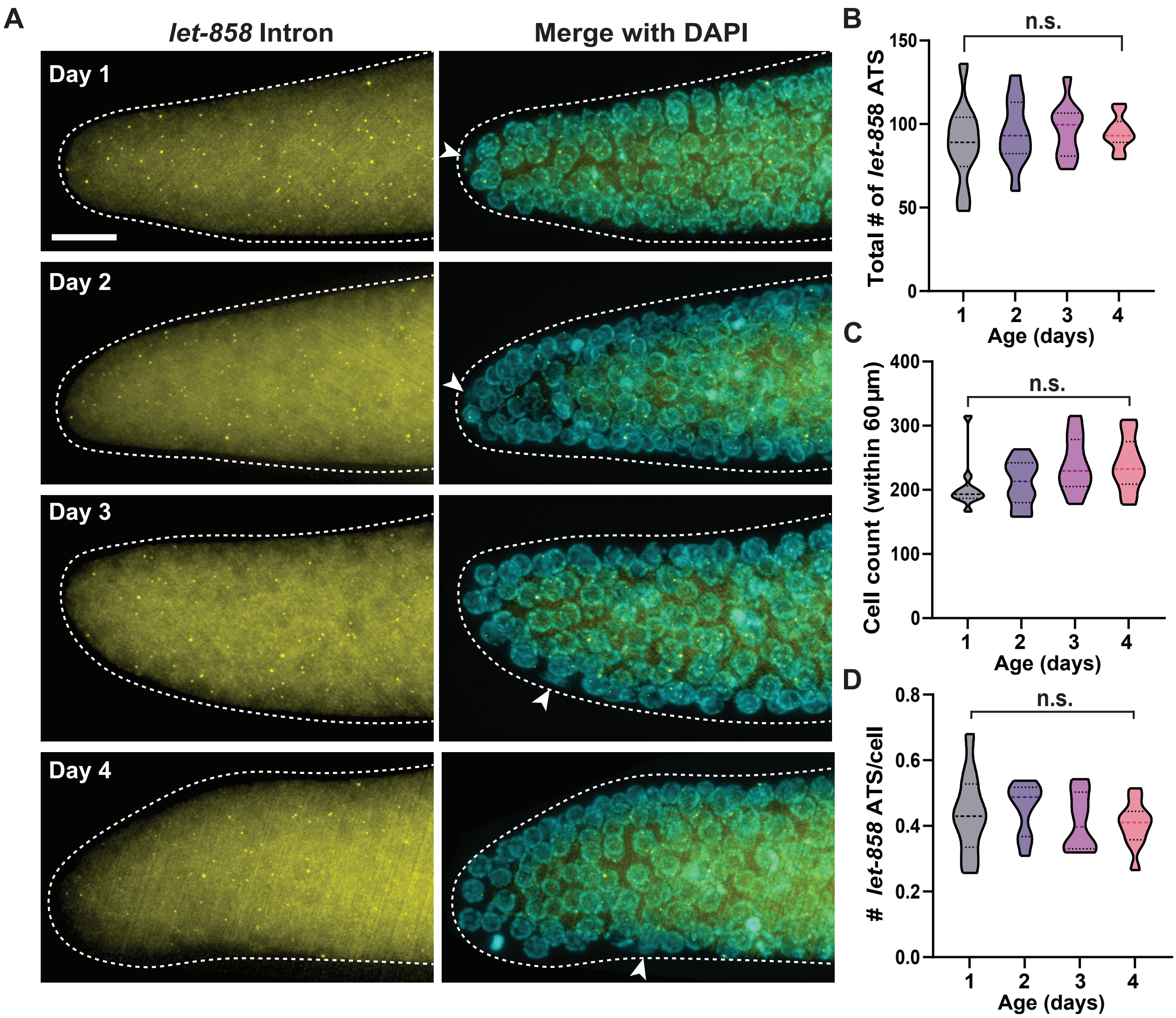

Fig. S2. (A) smFISH with a probe set targeting the introns of a Notch-independent gene, *let-858*, from Day 1- 4. White arrowhead: DTC/niche nucleus. White dashed line: the outline of the gonad. Scale bar: 10 µm. (B-D) The total number of *let-858* ATS within a gonad (B) the total number of germ cells in the distal gonad (0-60 µm from the distal end) (C) or the number of *let-858* ATS in each cell (D) was scored. n=10 gonads for each aging stage.

Biology Open • Supplementary information

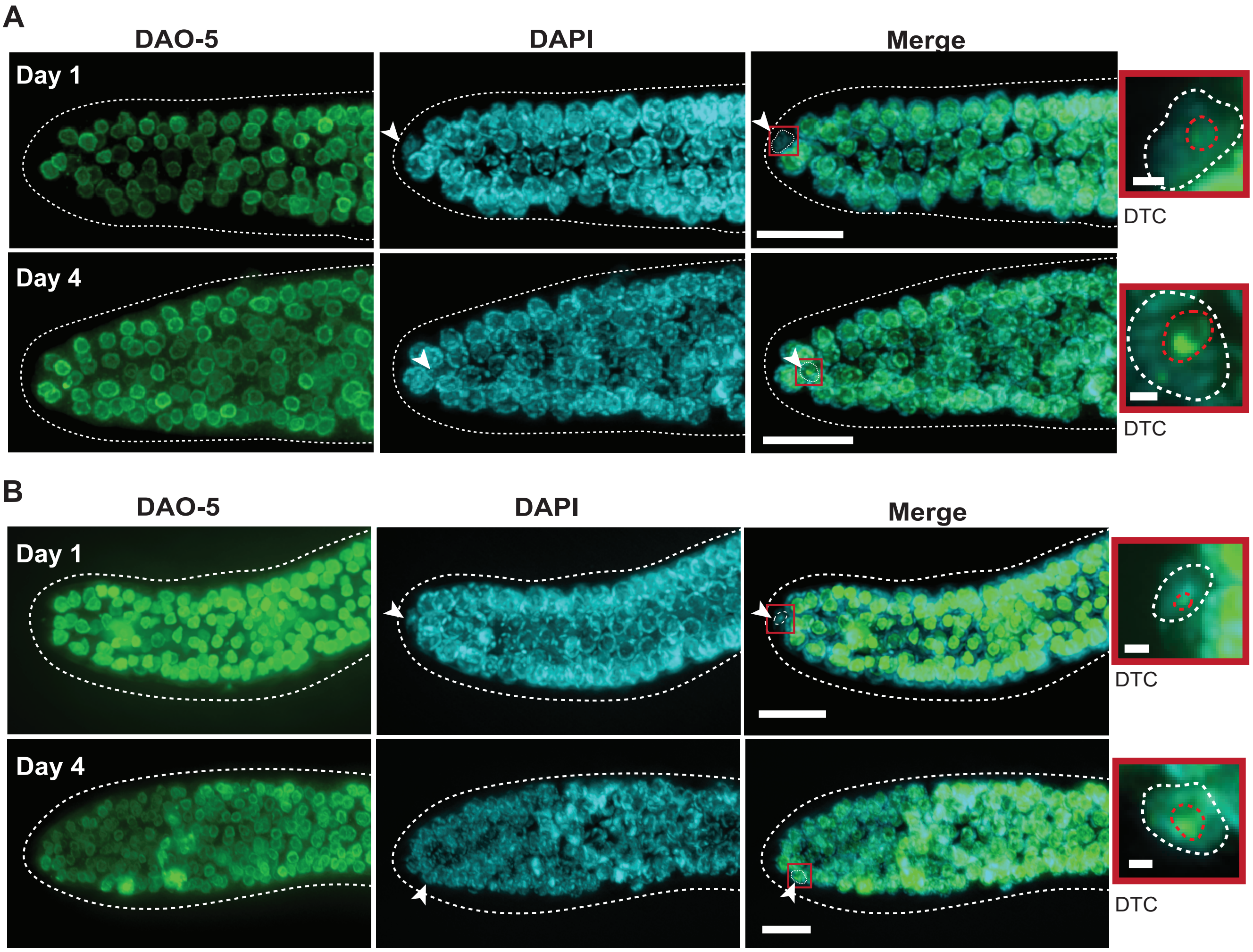

Fig. S3. (A-B) Left: maximum z-projection of DAO-5 staining and DAPI signal. DAO-5 staining visualizes the nucleolus whereas DAPI marks DNA. White arrowhead: DTC/niche nucleus. Scale bar: 10 µm. Right: the red boxed regions are zoomed in. White dashed line in the zoomed images: outline of the DTC/niche nucleus. Red dashed line: outline of the DTC/niche nucleolus. Inlet scale bar: 1 µm.

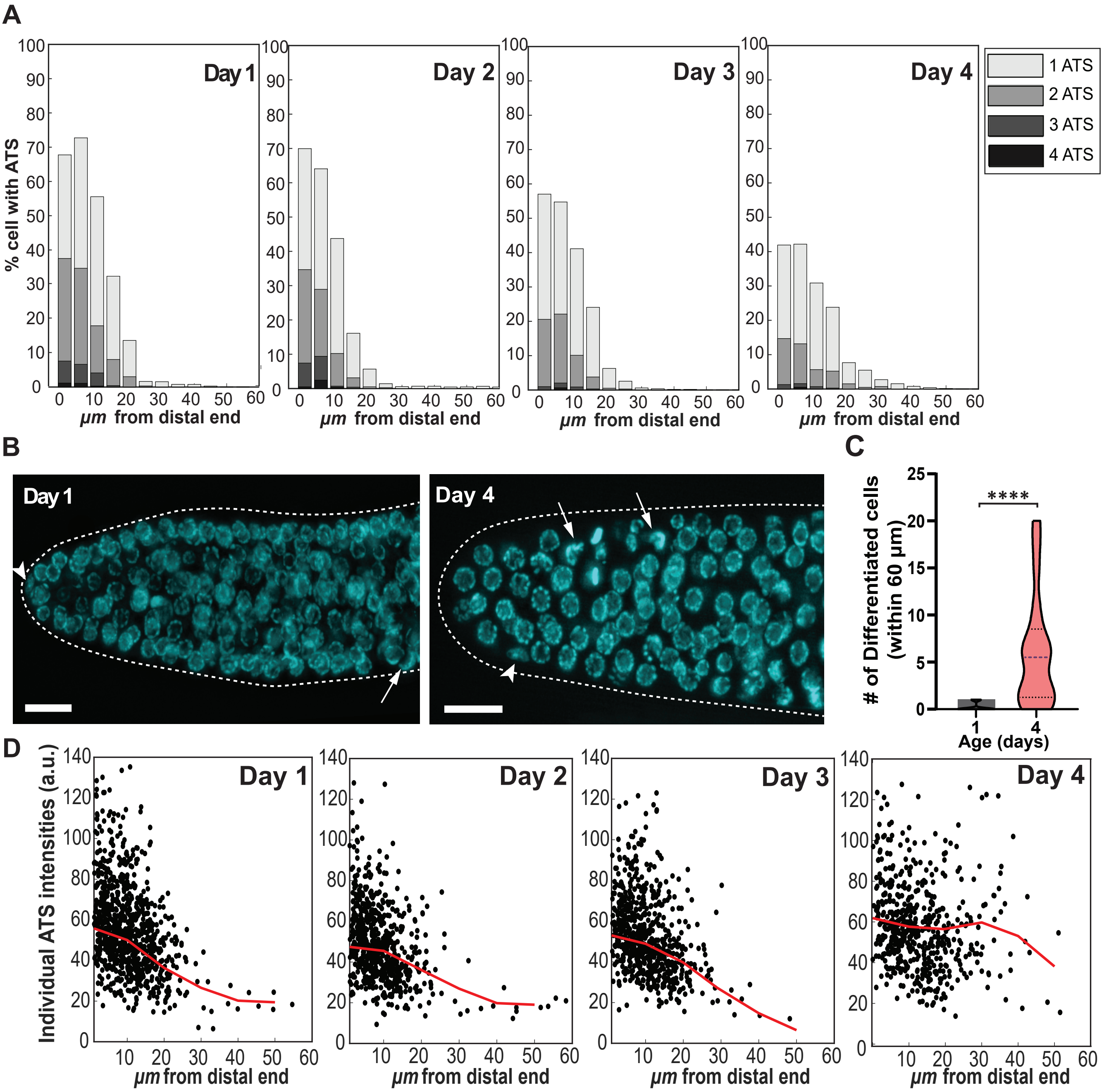

Fig. S4. (A) The percentage of germ cells containing at least one *sygl-1* ATS as a function of the distance from the distal end. Each bar is also separated by the number of *sygl-1* ATS in a cell, from 1 ATS per cell to 4 ATS per cell. n=27, 25, 26, and 24 gonads for Day 1, 2, 3, and 4 respectively. (B) DAPI staining at the distal gonad in Day 4. The arrow indicates nuclei at meiotic prophase or at pachytene, morphologically distinct from the miotic nuclei, an indication of premature germ cell differentiation. White arrowhead: DTC/niche nucleus. A partial Z-project is shown. Scale bar: 10 µm. (C) The number of differentiated meiotic cells found within 60 µm from the distal end of Day 1 and 4. n=20 gonads for each aging stage. (D) The Individual *sygl-1* ATS intensities are plotted against the distance from the distal end of Day 1, 2, 3, and 4. Each dot represents one *sygl-1* ATS. n=931, 661, 660, and 550 ATS for Day 1, 2, 3, and 4 respectively.

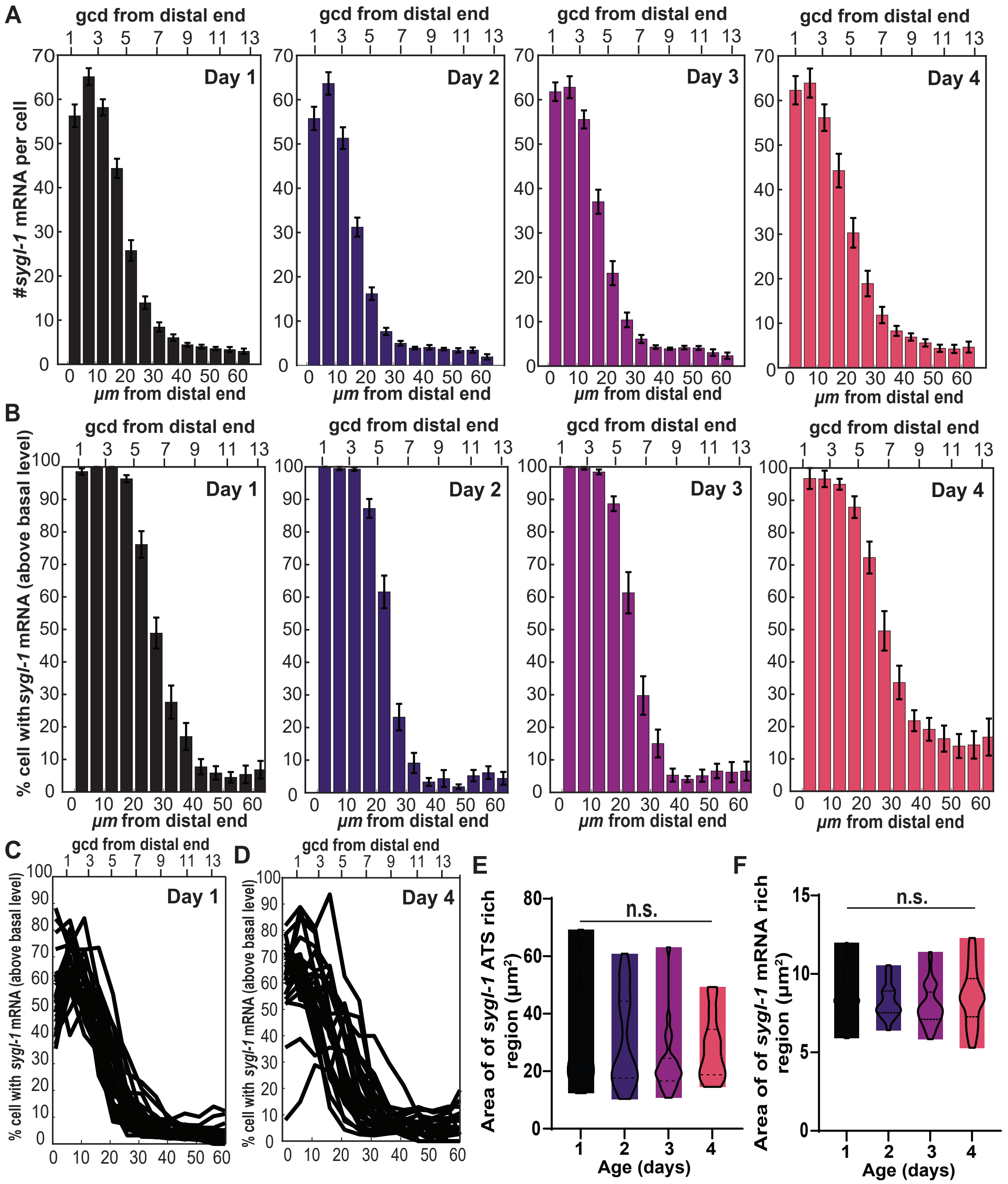

Fig. S5. (A) The total number of *sygl-1* mRNAs per cell as a function of distance from the distal end (B) The percentage of cells containing *sygl-1* mRNAs above the basal level, which is an average mRNA count in a cell at 40-60 µm from the distal end, where *sygl-1* is not actively transcribed by Notch signaling (see Materials and methods). (A-B) n=27, 25, 26, and 24 gonads for Day 1, 2, 3, and 4 respectively. (C-D) Line plots of the percentage of cells containing with *sygl-1* mRNA above basal level against the distance from the distal end of individual gonads overlaid at Day 1 (C) or Day 4 (D). (E) The size of the *sygl-1* ATS rich region, estimated by the distance between the distal end and *sygl-1* ATS-containing cells located most proximally. (F) The size of the *sygl-1* mRNA-rich area. (E-F) n=45 for all stages.

Biology Open • Supplementary information

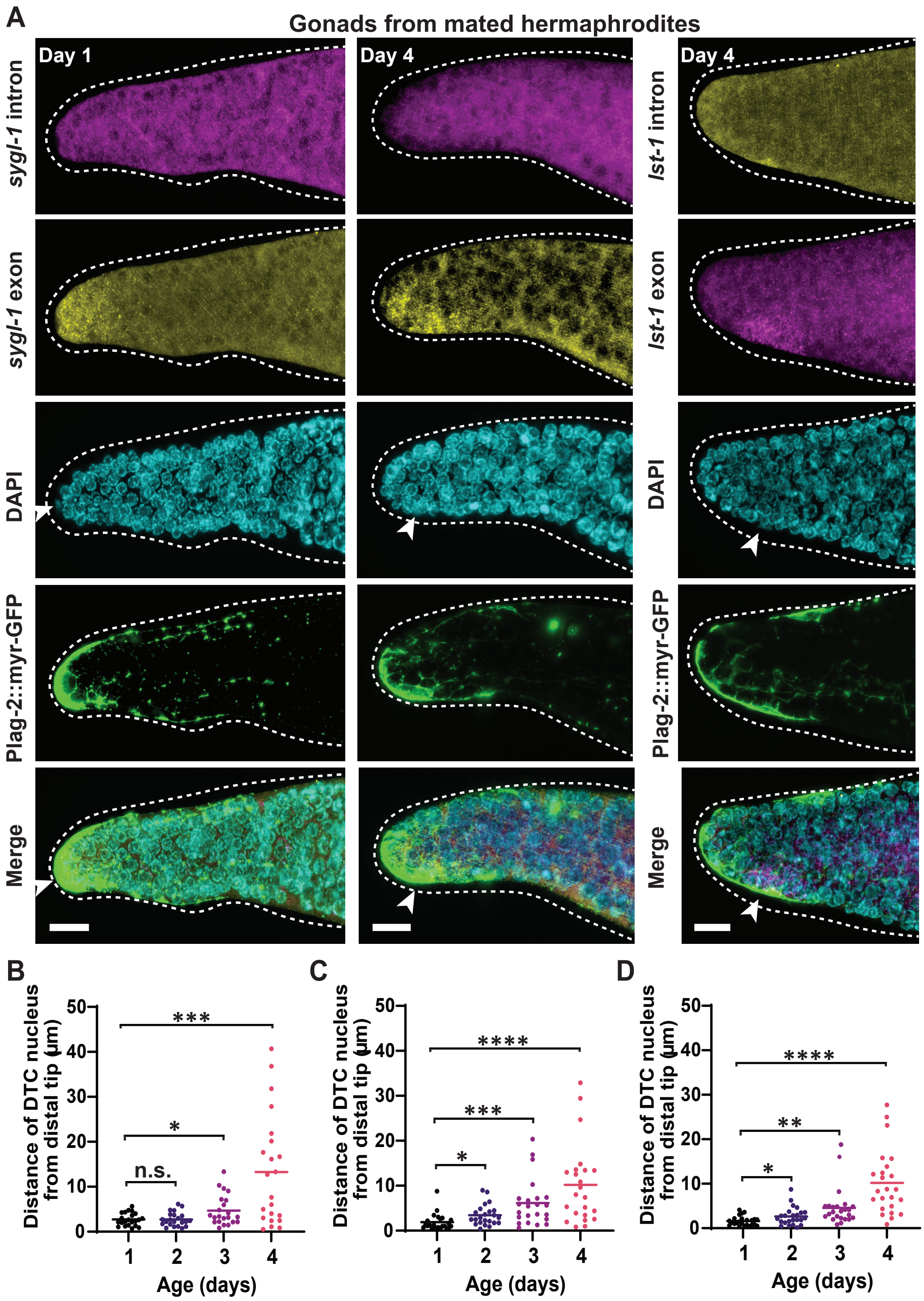

Fig. S6. (A) *sygl-1* and *lst-1* RNAs, DAPI signal, and DTC/niche plasma membrane are visualized in mated hermaphrodites at Day 1 (left), *sygl-1* Day 4 (middle), or *lst-1* Day 4 (right). RNAs are visualized by smFISH as shown in Fig.2 and DTC membrane is visualized by myristoylated GFP driven by *lag-2*. Proximal signal is present due to high background for smFISH/IF co-staining. Images are maximum z-projected. White arrowhead: DTC/niche nucleus. Scale bar: 10 µm. (B-D) Plots of individual replicate of the amount of nuclear shift measured by the Euclidean distance between the DTC/niche nucleus and the distal end of the gonad. Mean shown by solid line. (B) n= 22 gonads for all ages. (C) n= 23 gonads for all ages. (D) n= 24 gonads for all ages.

Biology Open • Supplementary information

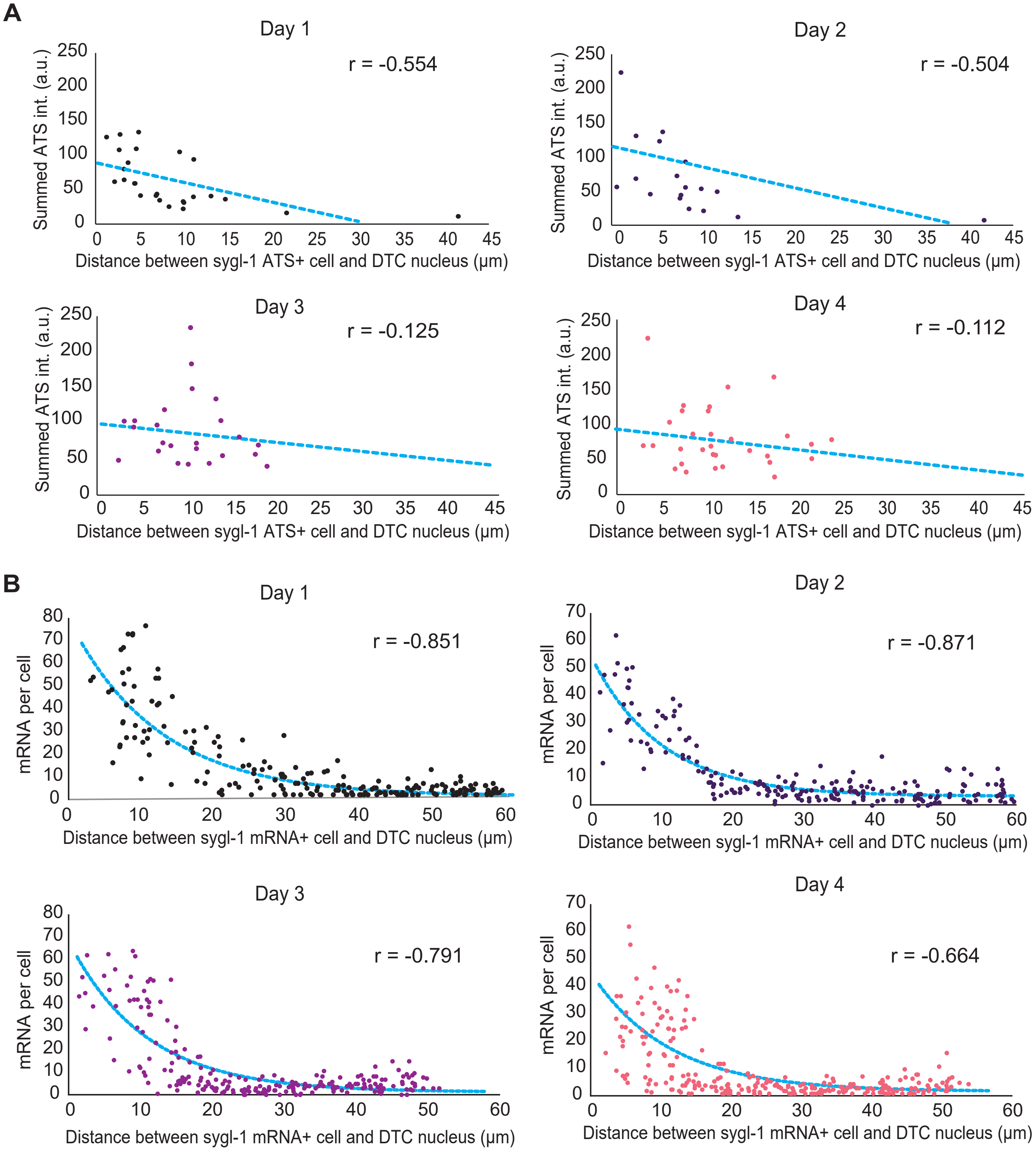

Fig. S7. (A-B) The summed *sygl-1* ATS intensities in each cell (A) or the number of *sygl-1* mRNAs per cell (B) of an individual gonad are plotted against the distance between the corresponding cell and the DTC/niche nucleus. ‘r’ indicates Pearson’s correlation coefficient. The blue dashed line indicates the line fitting model to calculate Pearson’s r value.

Biology Open • Supplementary information

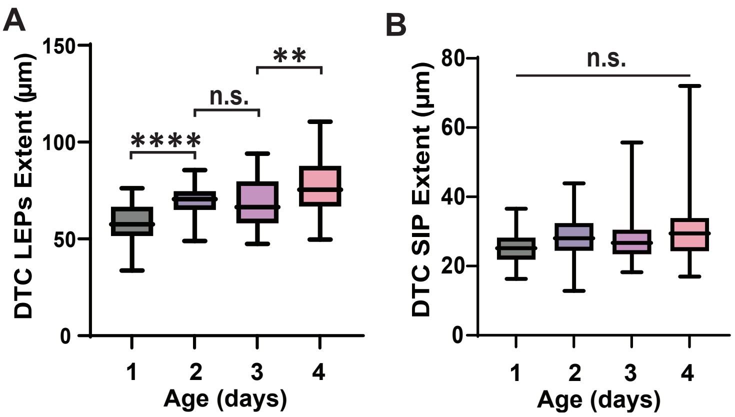

Fig. S8. (A) The length of the long external process (LEP) is measured in all ages. (B) The distance between the distal end and the most proximally located SIP is plotted, estimating the SIP extent in the gonad in all ages. (A-B) n=30, 30, 30, and 29 gonads for Day 1, 2, 3, and 4 respectively.
